# Influence of *Pholiota* *adiposa* on gut microbiota and promote tumor cell apoptosis properties in H22 tumor-bearing mice

**DOI:** 10.1038/s41598-022-11041-x

**Published:** 2022-05-21

**Authors:** Xiao-yan Wang, Ying Zhang, Fang-fang Liu

**Affiliations:** 1 Academy of Traditional Chinese Medicine, Jilin Agricultural Science and Technology University, Jilin, China; 2 Academy of Medical, Changchun Sci-Tech University, Changchun, China

**Keywords:** Biochemistry, Biotechnology, Cancer, Immunology, Microbiology

## Abstract

Hepatocellular carcinoma (HCC) is a common type of cancer—prevalent worldwide—and one of the causes of cancer-related deaths. In this study, ethanol extracts from *Pholiota* *adiposa* (EPA) were used to identify possible targets for HCC treatment and their effects on intestinal microflora were analyzed. Methods: Male mice were randomly assigned to groups—the model group, cyclophosphamide (25 mg/kg/d), and EPA groups, in which the mice were categorized based on the different concentrations of each compound (100, 200, and 300 mg/kg/day). Relevant biochemical indicators were detected using ELISA, H&E staining, and TUNEL assay. Four tumor apoptosis-related proteins and genes, Cleaved Caspases, *BAX*, Bcl-2, and VEGF, were detected by immunohistochemical staining, western blotting, and RT-PCR. The total genomic DNA was obtained from the contents of the small intestine and colon and was sequenced. The V3 + V4 regions of bacterial 16 s rDNA (from 341 to 806) were amplified. Results: The tests revealed that EPA exhibited antitumor activity in vivo by promoting apoptosis and inhibiting angiogenesis. Moreover, EPA treatment could increase beneficial and decrease harmful microflorae. These results demonstrate that EPA may be a potential therapy for HCC.

## Introduction

The incidence of liver cancer is high worldwide, with the highest observed morbidity and mortality rates. The incidence of liver cancer has decreased in developed countries, while it continues to increase in developing countries^2^. The currently available treatment routes for cancer include surgery, radiotherapy, and chemotherapy, but the efficiency of these treatments is low and they have various adverse effects^3^. Therefore, it is necessary to develop or discover new low toxicity drugs for cancer treatment. The mouse hepatocellular carcinoma (HCC) cell line H22 is used extensively to establish an animal tumor model^4^, and cyclophosphamide (CTX) is mostly used as the positive control drug to investigate the antitumor effects of bioactive compounds^5^.

Drug therapy is a conventional strategy against cancer, and there are several antitumor drugs available in the market, such as CTX, paclitaxel, and oxaliplatin. Although chemotherapeutic drugs have good tumor inhibiting properties, they also have significant side effects, such as dysfunction of other organs or gastrointestinal problems^6^. Natural sources, especially medicinal fungi, may be considered as potential hypoglycemic agents because of their high efficacy, and low toxicity.

*Pholiota* *adiposa* is a famous edible mushroom found in Northeast Asia, and its fruit body has a better antitumor effect. *P.* *adiposa* is rich in polysaccharides, proteins, vitamins, and sterols^7^. The fruit body of *P.* *adiposa* is rich in medicinal properties and contains functional substances, such as polysaccharides, peptides, polyunsaturated fatty acids, ribonucleic acids, lectins, triterpenoids, adenine, and steroids^8^. Consumption of *P.* *adiposa* has potential benefits, including heat dissipation, and promotion and regulation of the human body^9^.

The relationship between intestinal microorganisms and the occurrence and development of diseases has attracted considerable attention. In the human body, the gut microbiota has important functions in metabolism, digestion, and intestinal barrier protection^9^. At present, development of molecular biology technologies, which affect intestinal microorganisms, has attracted the attention of researchers. And maintain the stability of the gut internal environment of the organism^9–11^, where in the relatively stable structure of the gut microbiota is of great significance to human health. Several factors affect the diversity of gut microbiota, such as obesity, diabetes, irritable bowel syndrome, and cancer^9,12^. In addition, antibiotic use and diet also have certain effects on the diversity of the gut microbiota^9,13^. Increasing evidence indicates that aberrant gut microbiota has a profound impact on the onset and progression of cancer development; therefore, understanding the diversity of bacterial flora in feces can help understand the changes in disease^9^.

Previous research has shown that *P.* *adiposa* has a good antitumor effect^8^. In this study, ethanol extract from the fruit body of *P.* *adiposa* (EPA) was obtained and analyzed using LS-MS liquid mass spectrometry to demonstrate the antitumor activity of EPA in an H22 tumor-bearing mouse model. We investigated the possible molecular mechanisms underlying the antitumor action of EPA. Additionally, we also investigated the influence of EPA on gut microbiota using feces of tumor-bearing mice and compared it with that of the normal control group, model group, and EPA control group mice.

## Results

### The results of LC–MS analysis

**Figure 1:**
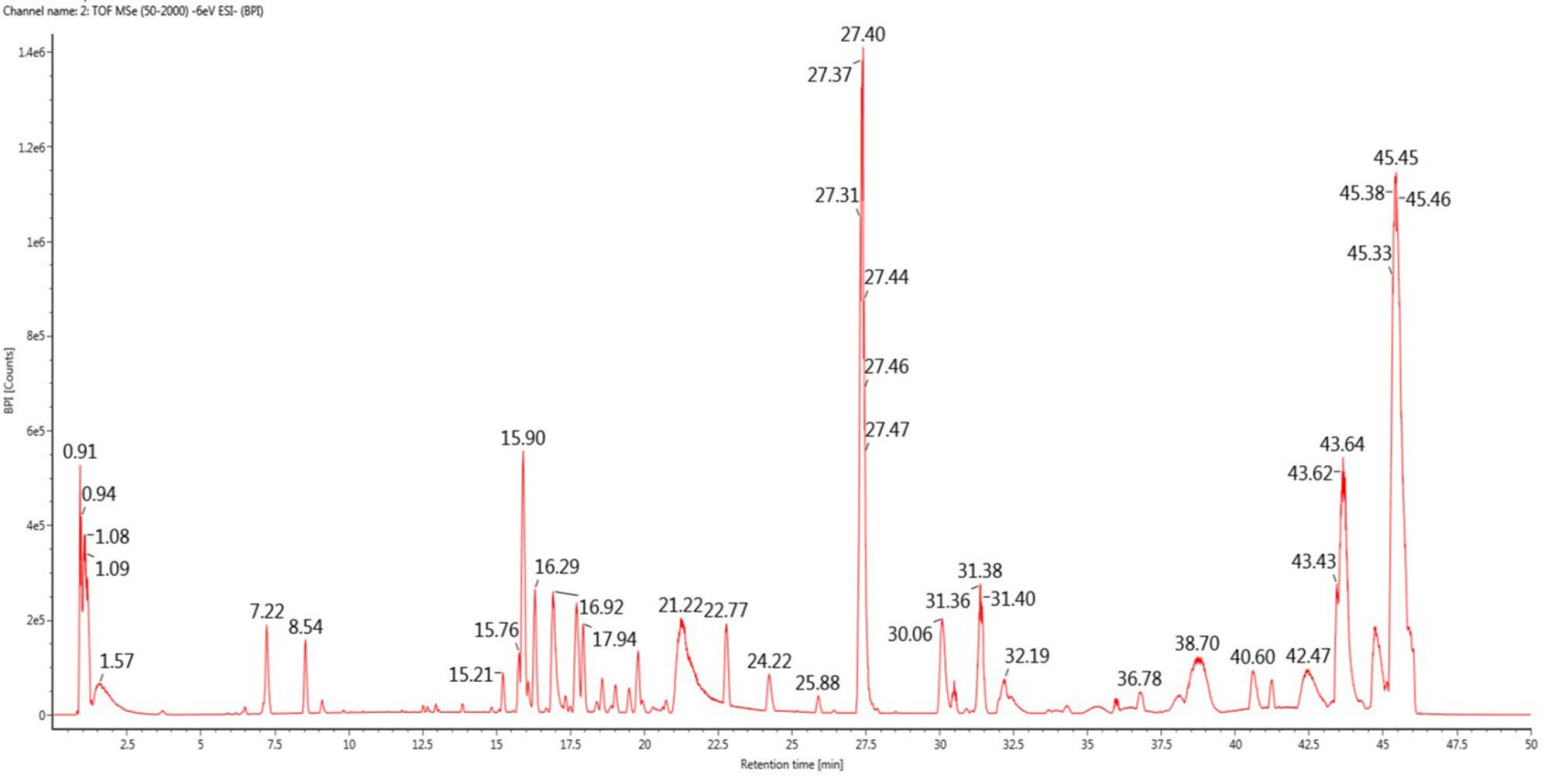
The total ion chromatogram of the extracted of Pholiota adiposa detected by LS-MS.

As shown in Fig. 1, in the present study, under optimal chromatographic and MS conditions^14,15^, 3 compounds were identified by comparing the retention times and matching the empirical molecular formulas with those of known saponins; six phenolic compounds, seven fatty acid compounds, three nucleotide compounds, five quinone compounds, six carbohydrate compounds, and four steroid compounds were identified, and sterol compounds, such as ergosterol peroxide, ergosta-4, 6, 8(14)22-tetraen-3-one, ergosterol, and (22E,24R)-ergosta-7, 22-dien-3β,5α,6β-triol, accounted for a high proportion (Table 1).

**Table 1:** Affiliation of key peaks of EPA.

RT (min)	CAS number	Component name	Chemical formula	Expected mass (Da)	Observed m/z	Mass error (mDa)	Percentage (%)
27.31	2061–64-5	Ergosterol peroxide	C_28_H_44_O_3_	428.32905	427.31985	− 0.51	0.799
27.40	19,254–69-4	Ergosta-4,6,8(14)22,-tetraen-3-one	C_28_H_40_O	392.30792	391.29748	− 0.71	0.3256
27.46	57–87-4	Ergosterol	C_28_H_44_O	396.64805	395.42196	− 0.82	0.35
30.06	14,858–07-2	(22E,24R) -ergosta-7,22-dien-3β,5α,6β-triol	C_28_H_46_O_3_	430.34471	465.31065	− 1.45	0.158

### Analyses of tumor weight, tumor inhibitory rate, and organ index

**Figure 2:**
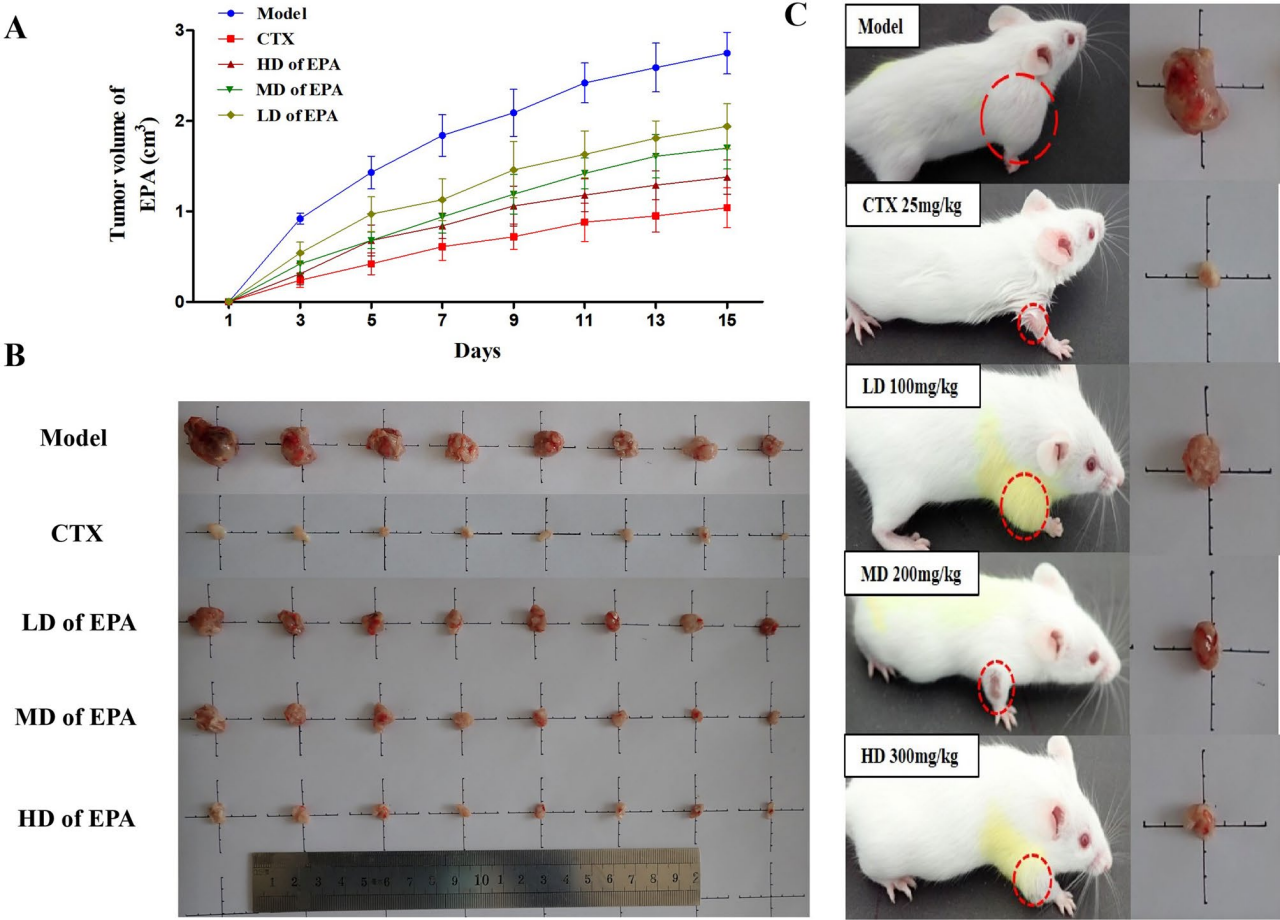
The effects of EPA on tumor H22 tumor-bearing mice.

The tumor volume growth curves are shown in Fig. 2. The tumor growth rate in the model group was more than 2.6 cm^3^ on Day 15. In contrast, CTX-treated and all EPA-treated groups showed obvious inhibition of tumor growth.

The organ index, tumor weight, and tumor inhibitory rate (TIR) are shown in Table 2. The CTX-treated group exhibited the highest TIR (92.42%). Moreover, the tumor weight in the EPA-treated groups significantly decreased in a dose-dependent manner, with tumor inhibitory rates of 79.70%, 84.55%, and 87.28%. The spleen and thymus indices were significantly lower in the CTX-treated group than in the normal group, and the thymus was severely atrophic (*p* < 0.05). The EPA-treated groups showed significant improvements, and there was a protective effect on the spleen and thymus.

**Table 2:** Effect of EPA on tumor weights and tumor inhibition rate.

Groups	Dose	Organ index (%)		Tumor weight (g)	Tumor Inhibitory rate (%)
		Thymus	Spleen		
Normal		5.75 ± 1.12**	5.62 ± 1.95**	–	–
Model		4.16 ± 1.37	10.86 ± 1.47^##^	3.30 ± 0.72	–
CTX	25mmg/kg	2.42 ± 0.48**^##^	2.73 ± 1.24**^##^	0.25 ± 0.12**	92.42%
LD of SLM	100 mg/kg	4.93 ± 1.25**	8.63 ± 1.31**^##^	0.67 ± 0.25**	79.70%
MD of SLM	200 m g/kg	4.87 ± 0.94**	7.54 ± 1.09**^##^	0.51 ± 0.18**	84.55%
HD of SLM	300 mg/kg	4.58 ± 1.19*	6.73 ± 1.36**^#^	0.42 ± 0.13**	87.28%

The values are presented as mean ± SD. n =10, *p < 0.05, **p < 0.01 compared with the Normal group, #p < 0.05, ##p < 0.01 compared with the Model group.

### Analysis of the expression of serum cytokines and VEGF in mice

ELISA was used to investigate the effects of EPA on serum cytokine production, including TNF-α, IFN-γ, IL-2, AST, BUN, and IL-6, in H22 tumor-bearing mice. Angiogenesis has become an important therapeutic target for many tumors and is an essential process for tumor growth and metastasis^10,16^.

**Figure 3:**
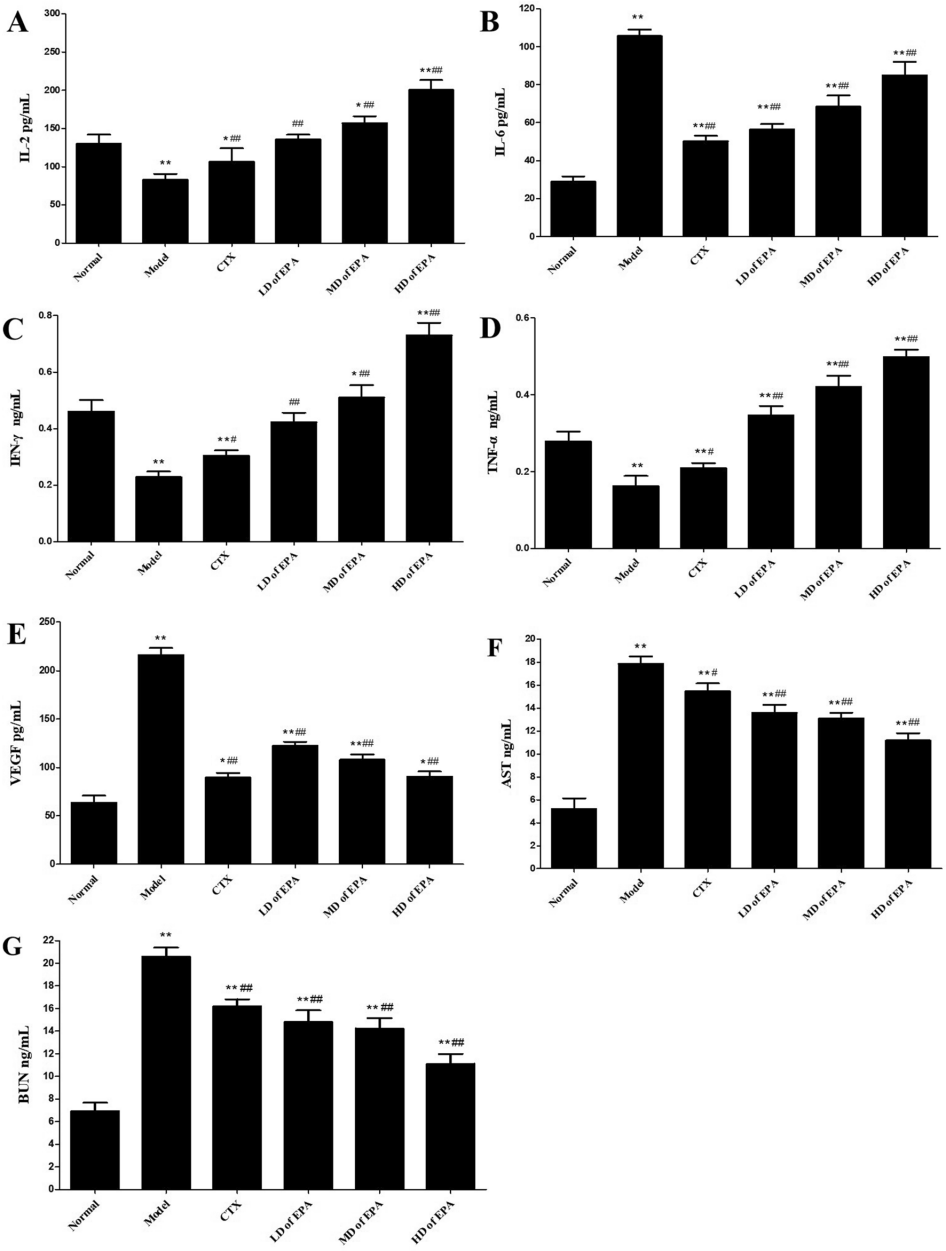
Effects of EPA on levels of serum cytokines, including, IL-2 (**A**), IL-6 (**B**), IFN-γ (**C**), TNF-α (**D**), VEGF (**E**), AST (**F**) and BUN (**G**).

As shown in Fig. 3, compared with the levels in the normal and model groups, the serum levels of IL-2, IL-6, IFN-γ, and TNF-α significantly increased in the EPA-treated HD group (*p* < 0.05). VEGF levels were significantly decreased in the CTX-treated and EPA-treated groups. The AST and BUN levels in the model group were significantly higher than those in the normal control group (*p* < 0.05). The values of these two parameters significantly decreased in the EPA-treated groups compared to those in the model control group (*p* < 0.05). The AST levels of mice in the CTX-treated group were significantly higher than those of the model group (*p* < 0.05).

### Morphological analysis after treatment with EPA

**Figure 4:**
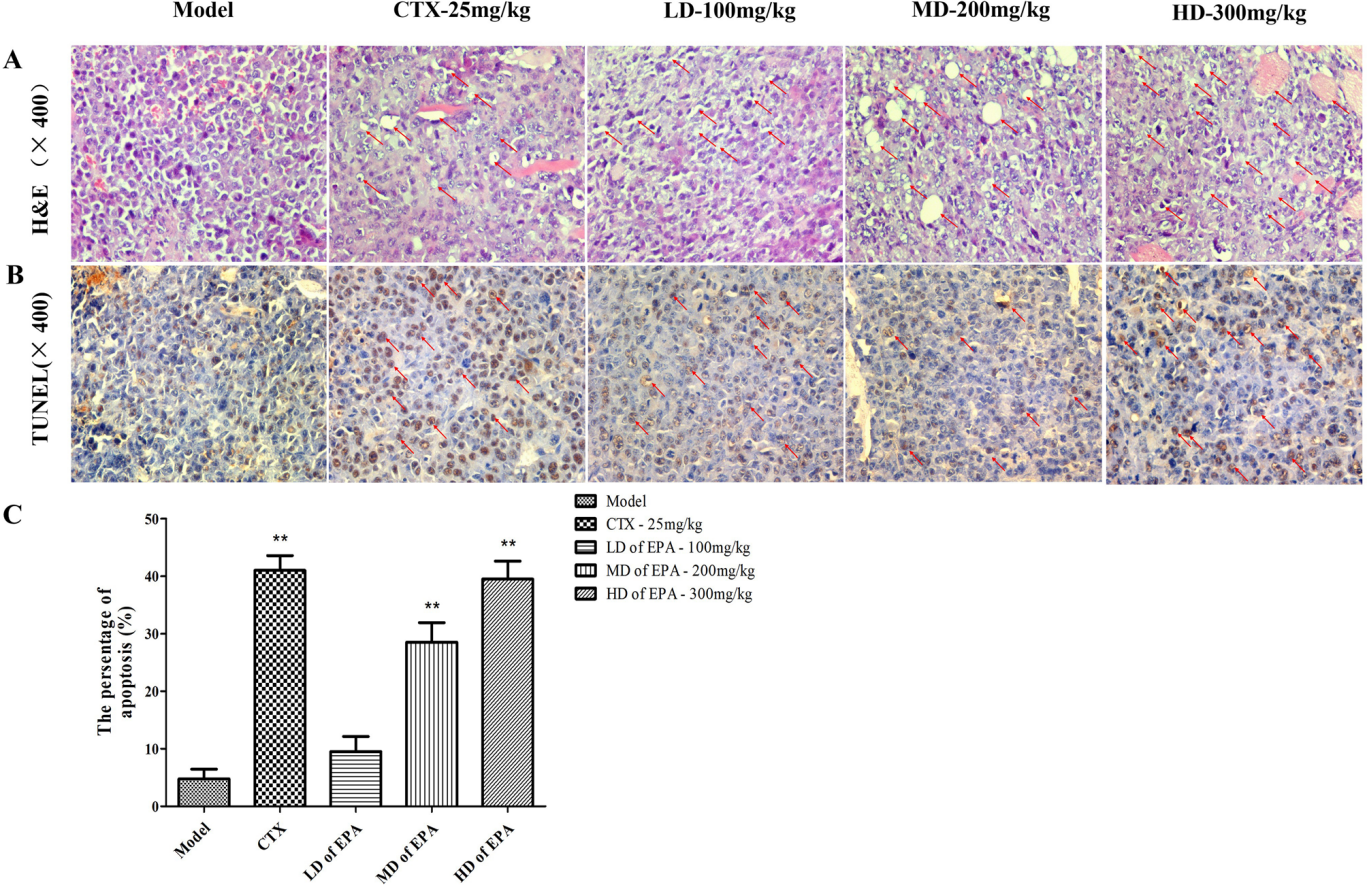
Histological examination of the morphological changes in tumors from H22-bearing mice. Tumor tissues stained with H&E (**A**) (400 ×) TUNEL assay (400 ×) (**B**,**C**).

Histopathology was evaluated using hematoxylin and eosin (H&E) staining of the tumor. As shown in Fig. 4A, tumor cell in the model group s were plump and intact, with large nuclei. After treatment with EPA and CTX, the tumor cells exhibited a large necrotic region and were stained darker, which indicates that the significant antitumor efficacy of EPA in H22 tumor-bearing mice involved inducing cell death.

The TUNEL assay results are shown in Fig. 4B,C, and cells with brown granules were considered positively stained^10,17^. The percentage of positive cells in the model group was 5%; therefore, the amount of tumor cell apoptosis was minimal. However, the CTX-treated group exhibited the highest percentage of positive cells (44%). The EPA-treated groups showed an increase in the number of cells undergoing apoptosis, with values of 12%, 28%, and 42%. Moreover, the mice in the HD and EPA-treated group showed a positive cell percentage similar to that of the CTX-treated group.

### Effects of EPA on the expression of apoptosis-related proteins and VEGF

**Figure 5:**
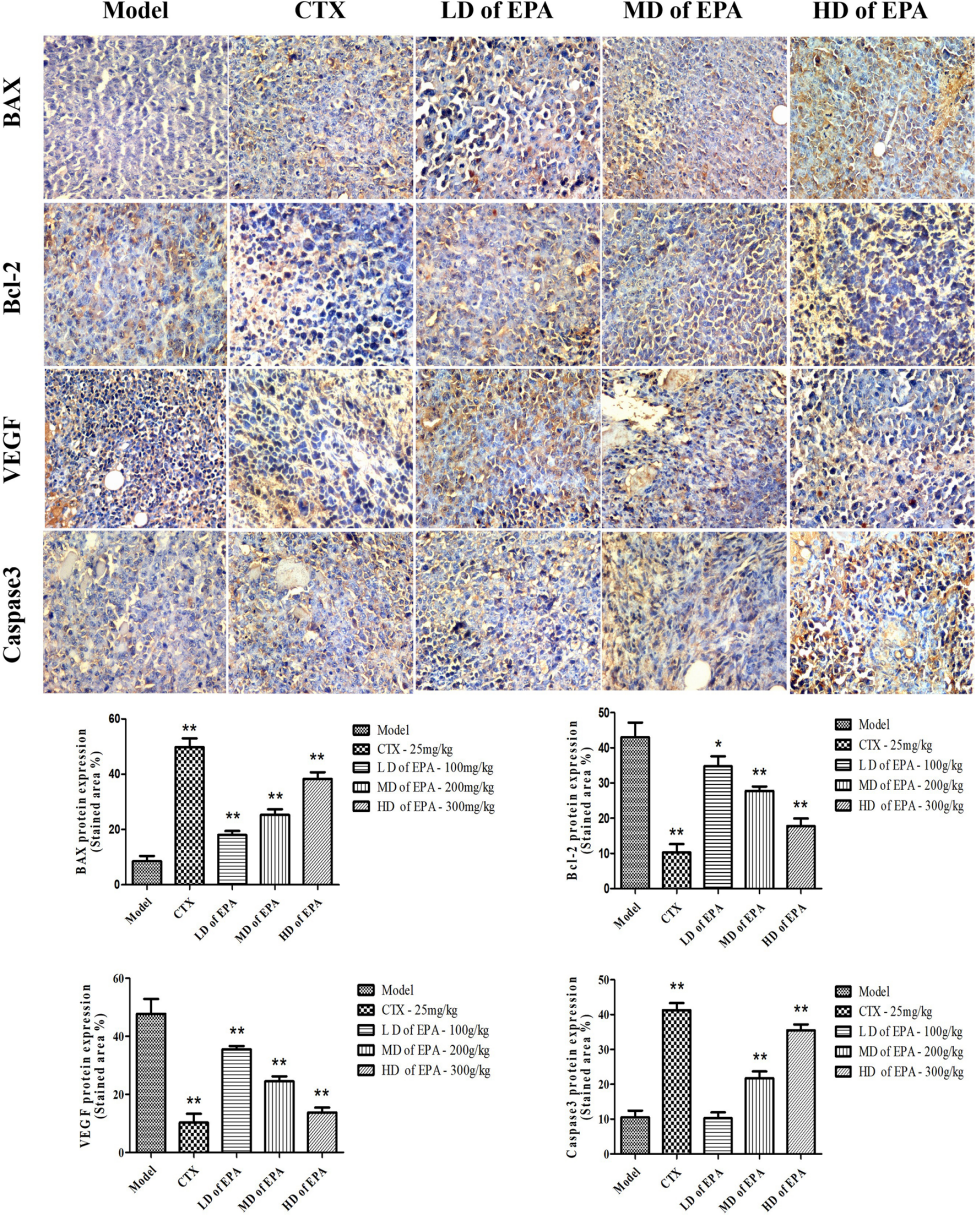
Immunohistochemical anylisis of tumor tissues and stained area of tumor tissues.

Based on the results of previous studies^18^, immunohistochemical analysis was performed on the proapoptotic factor BAX, the antiapoptotic factor Bcl-2, and cleaved caspase 3 to determine the expression of apoptosis-related proteins, as shown in Fig. 5. BAX and cleaved caspase 3 expression intensities increased, while Bcl-2 expression decreased in the EPA-treated groups in a dose-dependent manner. Therefore, the ratio of Bcl-2 to BAX decreased significantly. The vascular endothelial growth factor (VEGF) level in the EPA-treated groups significantly decreased in a dose-dependent manner, and VEGF expression was lowest in the CTX-treated group.

**Figure 6:**
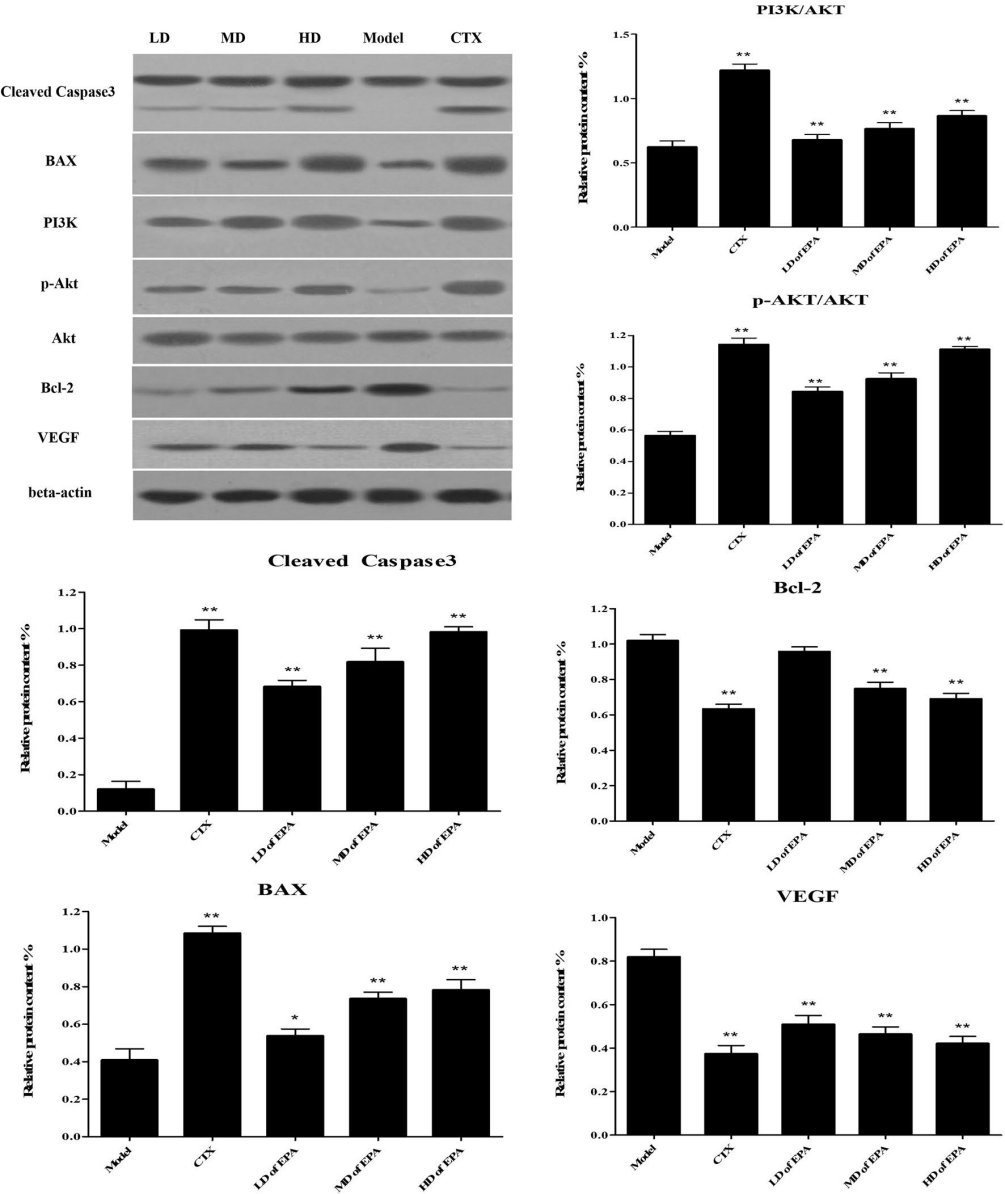
Relative protein expression of BAX, Bcl-2, Caspase3, AKT, p-AKT,PI3K and VEGF in tumor tissue.

In addition, western blotting was used to analyze the expression of Bcl-2, BAX, cleaved caspase 3, and VEGF. As shown in Fig. 6, all EPA-treated groups showed increased BAX and cleaved caspase 3 protein expression and decreased Bcl-2 and VEGF protein expression; these results were consistent with the results of the immunohistochemical analysis. However, the expression of PI3K/AKT and p-AKT/AKT increased in a dose-dependent manner in the EPA-treated group.

### Results of the rank-abundance curves of microorganisms in samples

**Figure 7:**
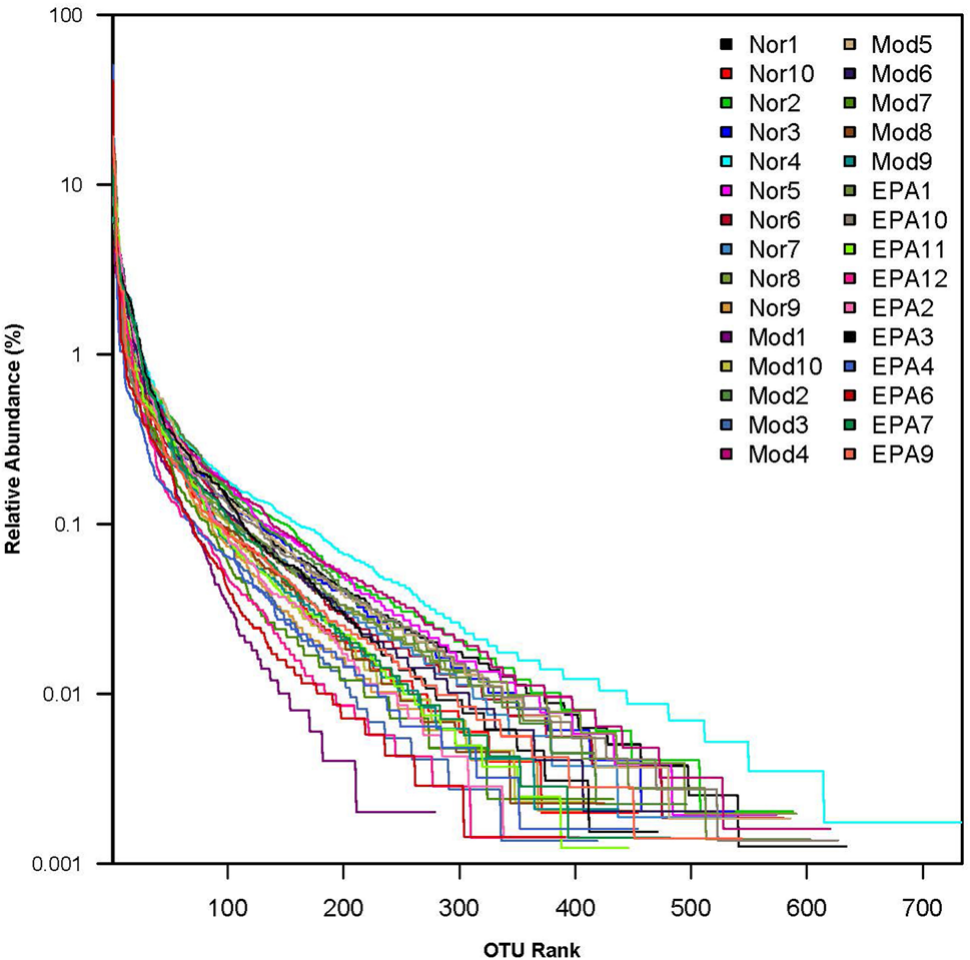
OTU Rank-Abundance curves. Different samples are represented by curves of different colors. The abscissa is the number rank sorted by OTU abundance, and the ordinate is the corresponding OTU abundance.

The rank-abundance curve is a method used to analyze the diversity of microorganisms. In this study, the operational taxonomic unit (OTU) rank-abundance curves showed the diversity in the samples, which can be explained by species richness and evenness^19^. In the horizontal direction, the wider the span of the curve is, the richer the species rank of the microorganism sample^20^. The OTU rank-abundance curve in this study showed that the abundance distribution of the samples was even, as it was flat and smooth in the vertical direction (Fig. 7). The richness and evenness of the samples shown in the rank-abundance curve indicate that the diversity of the samples was rational and reasonable.

### Dilution curves of microorganisms in the gut microbiota

**Figure 8:**
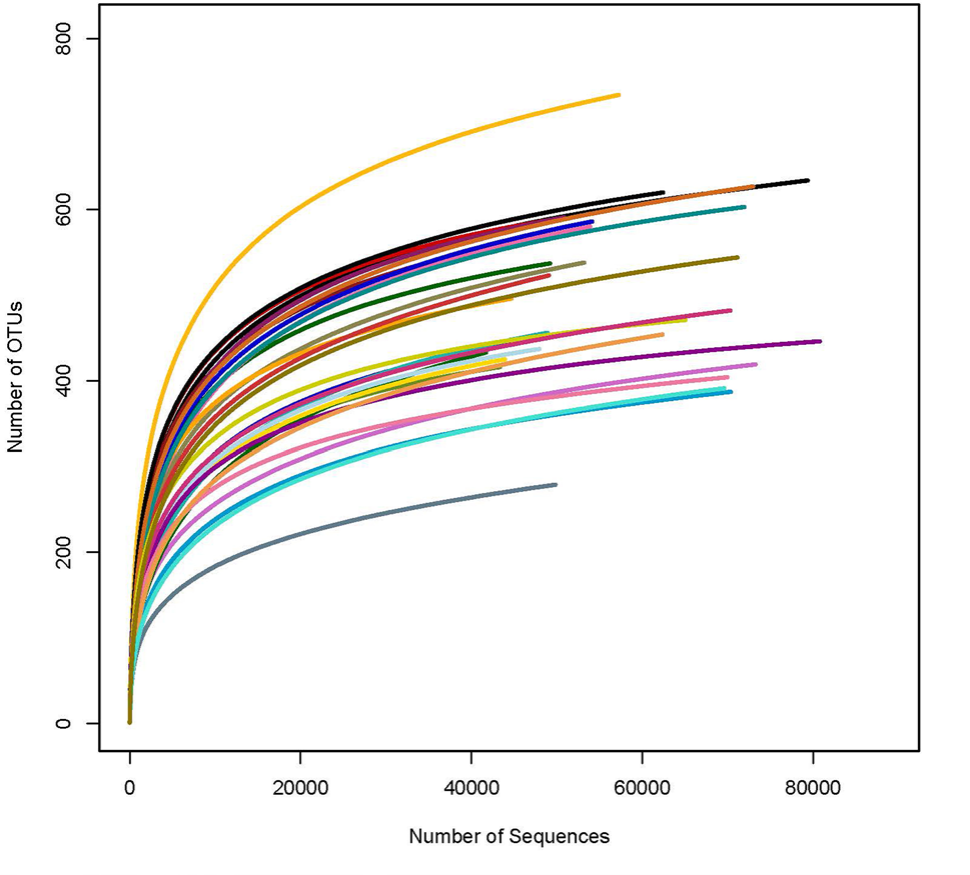
Dilution curve of the alpha diversity inde for species abundance of samples. The horizon axis represents the number of clean reads randomly extractrd from a sample, and the vertical axis represents the alpha.

The dilution curve was used to evaluate whether the sample sequencing depth was sufficient to cover all species of microbes and compare species richness among different sequenced samples. A flat curve indicates that the sequencing data are reasonable and reflect the structure of the entire community^21^. The number of sequences reached 5000, and the curves were in the plateau stage (Fig. 8). Therefore, the experimental data were deemed reasonable and reliable.

### Species accumulation curves of the gut microbiota

**Figure 9:**
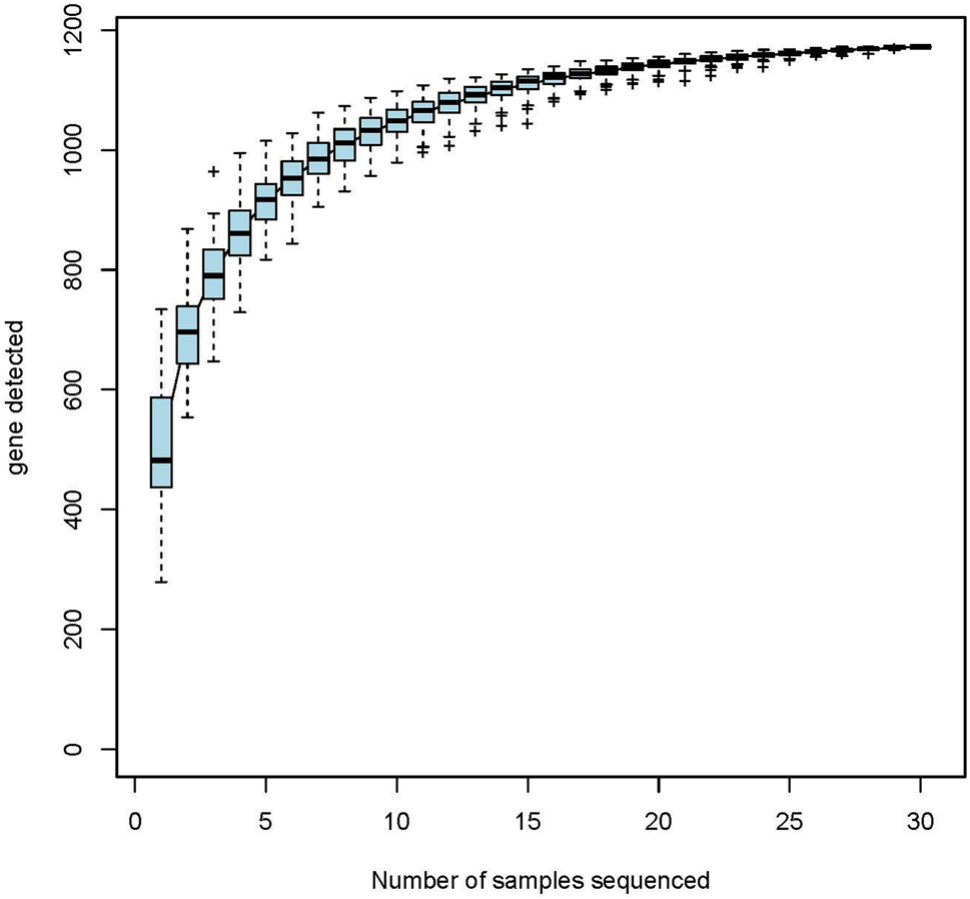
Species accumulation curves. Abscissa sample size ordinate OTU number after sampling.

The species accumulation curve is used to describe the increase in species with the increase in the number of samples and is an effective tool for investigating the composition and predicting the abundance of species in samples. This curve reflects whether the number of samples was sufficient. In this study, we mainly observed the end of the curve; if it showed a sharp upward trend, the number of samples was considered insufficient, while if the end of the curve tended to be flat, the number of samples was considered sufficient^22^. The end of the species accumulation curves of Specaccums tended to be flat in this study; therefore, the sampling was considered sufficient to carry out data analysis (Fig. 9).

### Results of alpha diversity analysis of the gut microbiota

**Figure 10:**
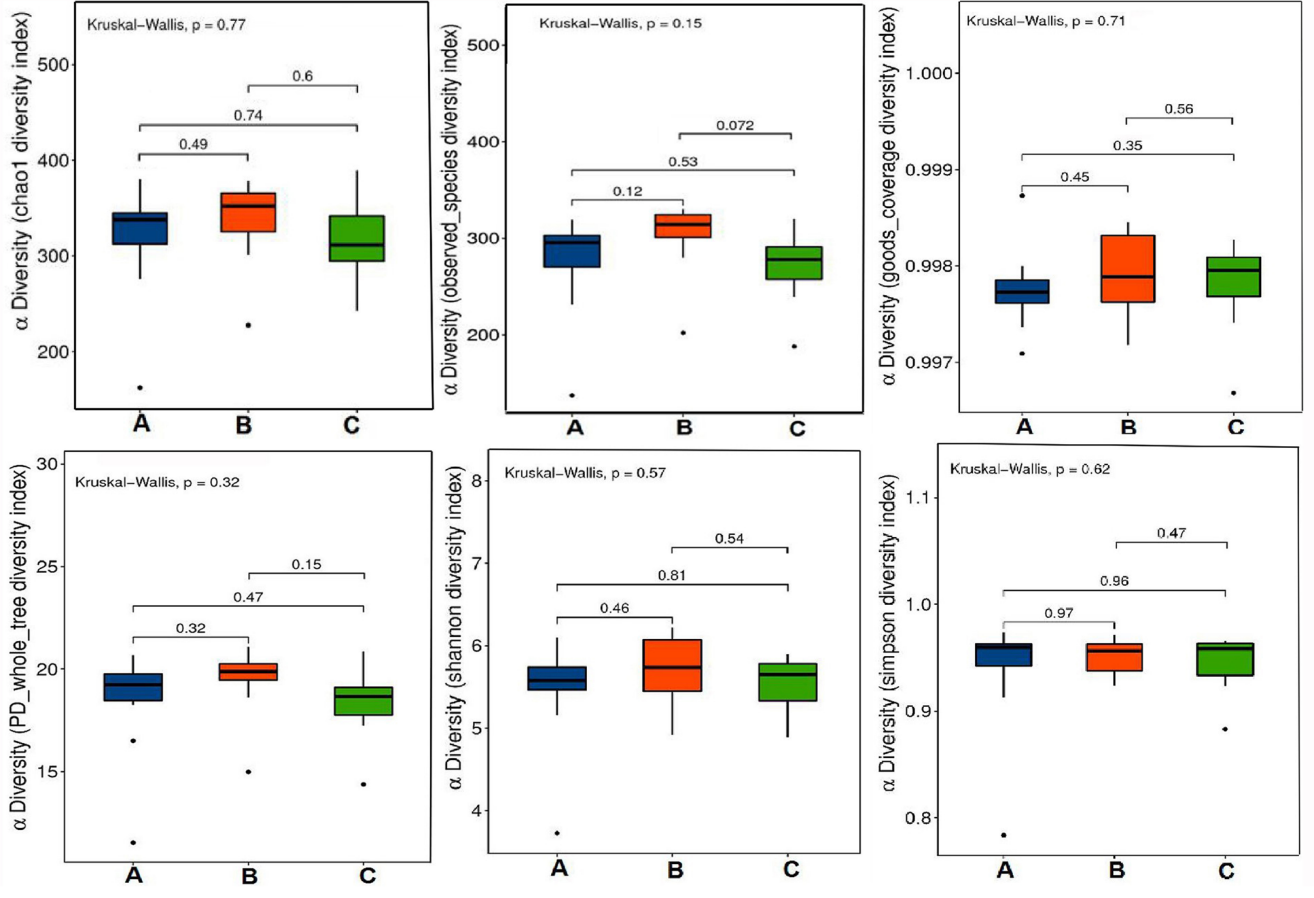
Alpha-diversity boxplots in Normal group, Model group and HD of SLM group, such as Chao1 (**a**), Observed (**b**), Goods_coverage (**c**), PD_whole_tree (**d**), Shannon (**e**), and Simpson indexes (**f**).

In this study, six indicators, including the Chao1 index, observed species index, PD_whole_tree index, goods-coverage index, Shannon index, and Simpson's index, were analyzed to assess the species diversity of individual samples (Fig. 10). There was no significant difference in flora abundance or diversity between the normal, model, and HD groups in the EPA-treated group.

### Results of beta diversity analysis of the gut microbiota

Beta diversity analysis was used to compare the differences in species diversity among intergroup samples, and the evolutionary distance between species was considered while calculating the results. The higher the index value, the greater the difference between the samples. In this study, the UniFrac results were divided into two parts: the weighted UniFrac, which considered the abundance of the sequence, and the unweighted UniFrac, which did not consider the abundance of the sequence. According to the statistical analysis of the differences of the clustered samples, the similarity of species composition among the samples was determined by calculating the distance between the samples^23^.

**Figure 11:**
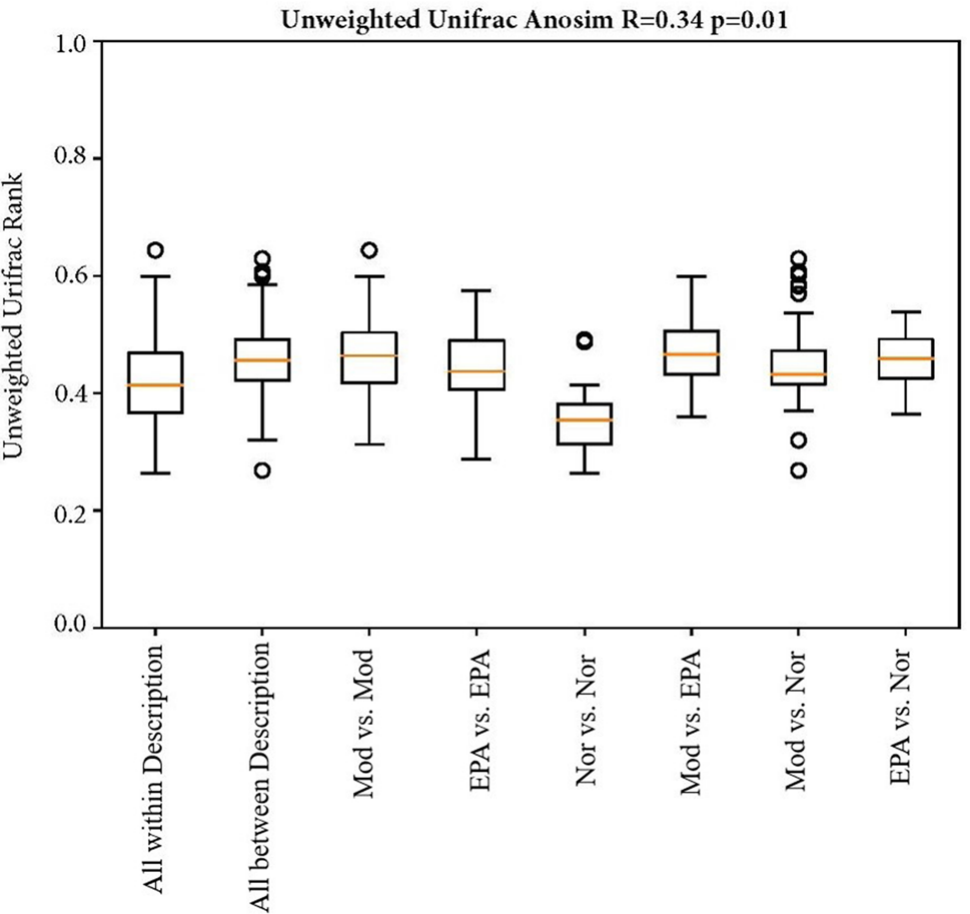
Anosim analysis of gut microbiota based on unweighted unifrac distance.

ANOSIM similarity analysis was used to determine the intergroup and intragroup differences, and the grouping was found to be meaningful (Fig. 11). The R value of the unweighted results was R = 0.34 > 0 and p = 0.001 < 0.05, indicating that the differences between the groups were significantly greater than those within the group (intragroup). Therefore, the result is reasonable, and the data are statistically significant. Thus, in this study, the gut microbiota in tumor-bearing mice tended to be normal after EPA treatment.

**Figure 12:**
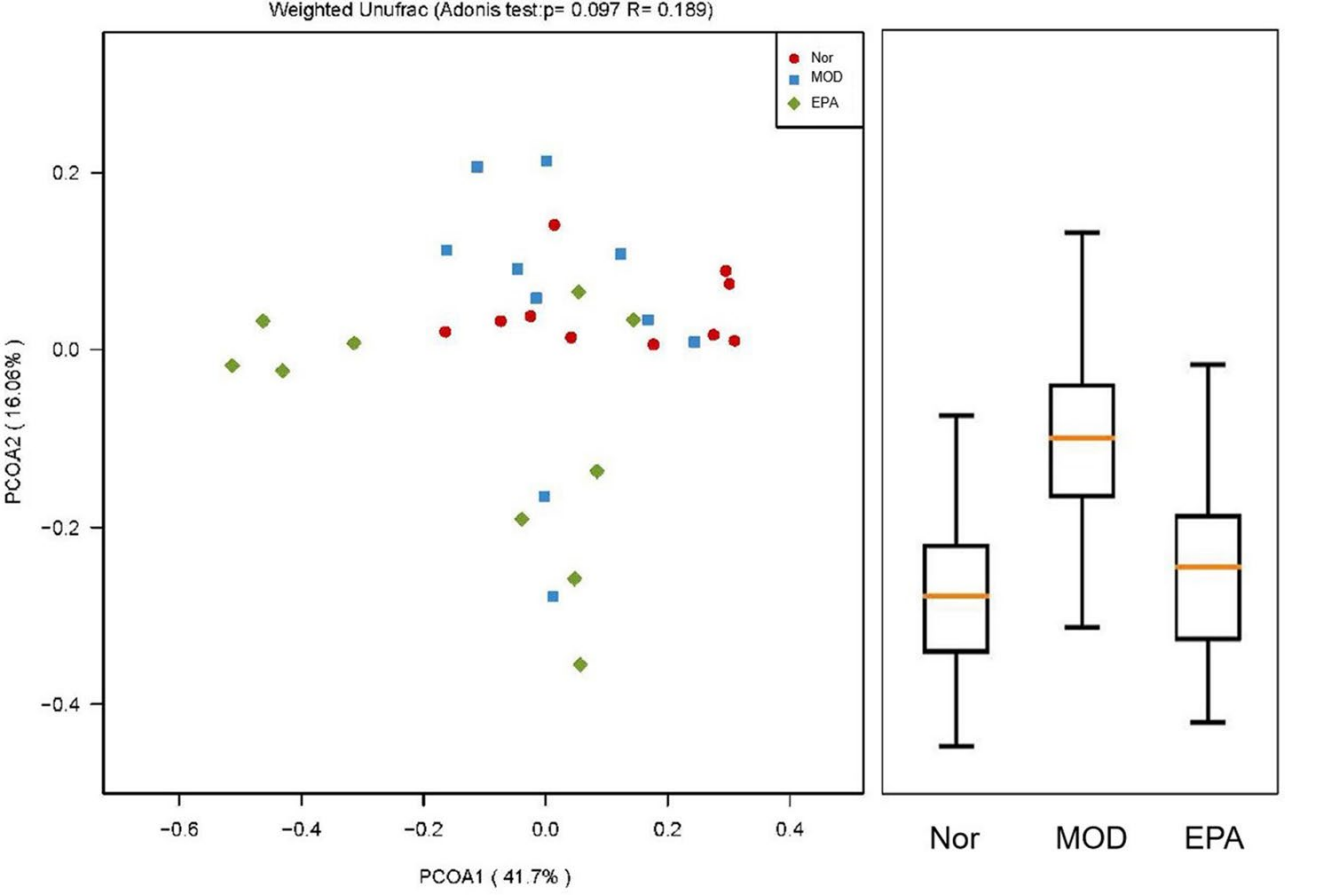
Principal coordinates analysis (PCoA) analysis of gut microbiota based on Weighted Unifrac distance. It illustrated the difference in the microbial composition among the samples.

Principal coordinates analysis (PCoA) was used to evaluate the differences between samples (Fig. 12)^24^. If the samples were in close proximity, the species compositions of the samples were similar. The EPA-treated group was closer in distance to the normal group, indicating that both groups had similar gut microbiotas.

### Difference Analysis of the Gut Microbiota

**Figure 13:**
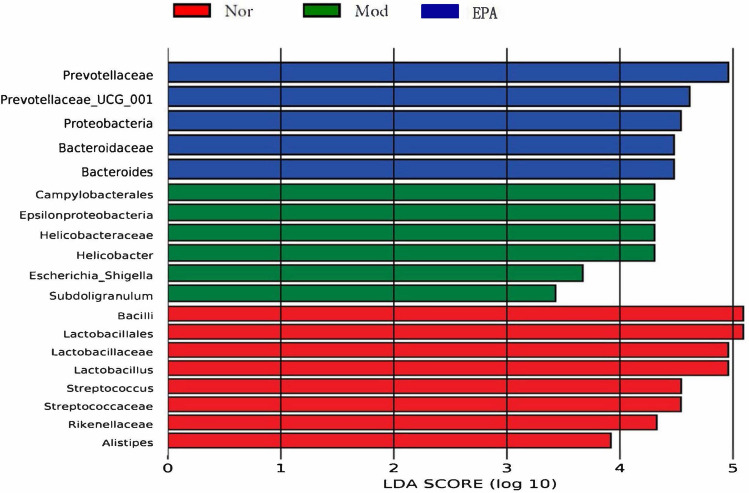
The LDA score obtained from LEfSe analysis of gut microbiota in different groups.

**Figure 14:**
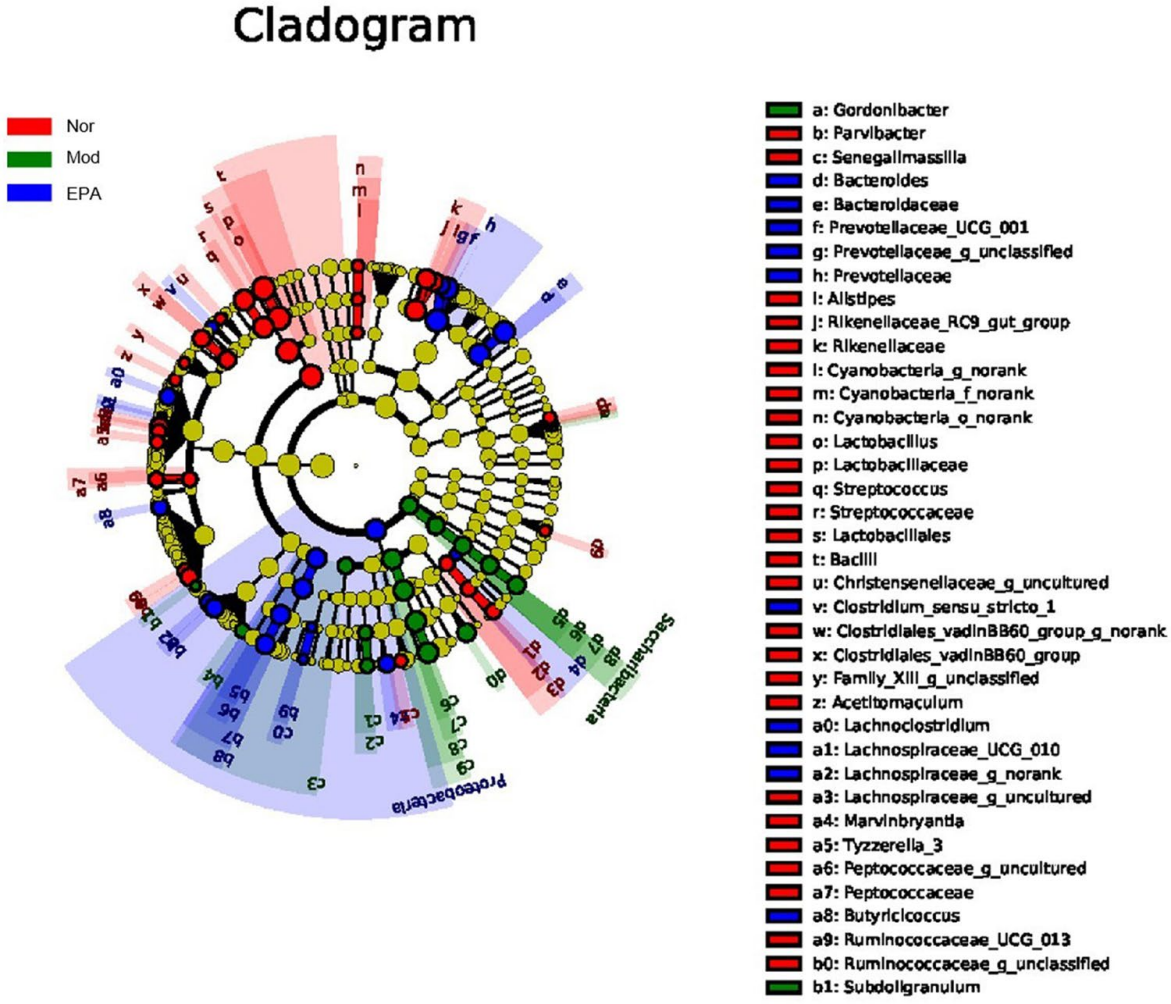
The LDA score obtained from LEfSe analysis of gut microbiota in different groups.

To emphasize the statistical significance and biological relevance, the LEFse analysis of the gut microflora was performed, and the results are presented quantitatively in an LDA Score column and a cladogram. This study aimed to estimate the impact of the abundance of each component and identify the significant difference effect on the division of samples^23^. The results showed significant differences in the gut microflora, such as *Lactobacillus*, *Streptococcus*, *Streptococcaceae*, *Rikenellaceae*, *Alistipes* (Nor), *Campylobacterales*, *Epsilonproteobacteria*, *Helicobacteraceae*, *Helicobacter*, *Escherichia*_*Shigella*, *Subdoligranulum* (Mod), *Prevotellaceae*, *Proteobacteria*, *Bacteroidaceae*, *Bacteroides*, *and* *Selenomonadales* (EPA) (Figs. 13 and 14).

### Species abundance in the gut microbiota

**Figure 15:**
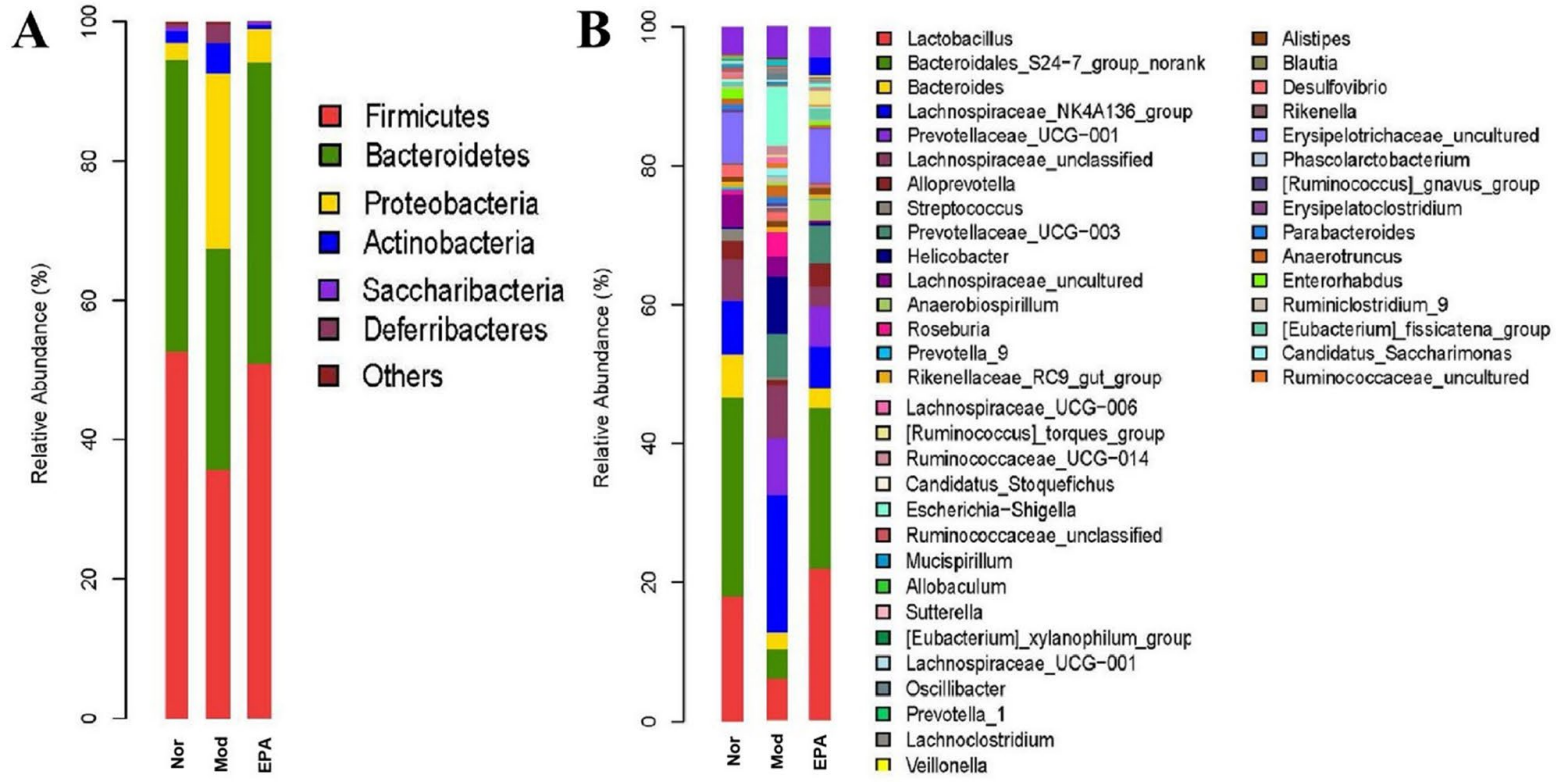
Taxonomic profiles of the fecal bacteria in the four groups at the phylum level (**a**) and genus level (**b**).

Histograms showing species profiling in the gut microflora of each sample were used to identify the species with higher relative abundances and individual samples at different classification levels (Fig. 15A and Table 3)^25^. In this study, the phylum level showed that *Firmicutes*, *Bacteroides*, and *Proteobacteria* accounted for more than 98% of the total microflora after the different treatments. Compared with that in the normal group, the abundance of *Bacteroidetes* and *Firmicutes* decreased, but *Proteobacteria* increased in the model group. In contrast, the abundance of *Bacteroidetes* and *Firmicutes* increased, but *Proteobacteria* decreased in the EPA-treated group and was close to that in the normal group. Thus, the trend in the two phyla in the microflora in the EPA-treated group trended more toward that in the normal group.

**Table 3:** The relative abundance of the main gut microbiota at the phylum level in four groups.

**Abundance (%)**			
	**A**	**B**	**C**
*Firmicutes*	53.63254	38.56821	51.84526
*Bacteroidetes*	37.78549	31.18542	39.85831
*Proteobacteria*	3.37934	24.44789	7.15324
Total	94.79737	94.20152	98.85681

Figure 15B shows the genus level of the gut microflora in the three groups. Based on abundance, the main groups were *Lactobacillus*, *Bacteroidales_S24 − 7_group_norank*, *Bacteroidales*, *Lachnospiraceae_NK4A136_group*, *Prevotellaceae_UCG − 001*, *Alloprevotella*, *Streptococcus*, *Alistipes*, *Prevotellaceae_UCG − 003,* and *Helicobacter*. Based on the changing trend in the abundance of the gut microflora, it can be concluded that tumor-bearing mice that were treated with EPA tended to be closer to those in the normal control group. Compared with that in the normal group, the abundance of beneficial microflora such as *Lactobacillus*, *Bacteroidales_S24 − 7_group_norank*, *Bacteroidales*, *Alloprevotella* and *Alistipes* in the model group decreased by 6.95%, 25.25%, 4.67%, 2.65%, and 0.68%, respectively; accordingly, compared with those in the model group, those in the EPA group increased by 10.67%, 19.94%, 1.34%, and 3.29%, respectively, which showed a significant reversion in the reduction in beneficial microflora in the model group. In contrast, compared with that in the normal group, the abundance of the harmful microflora *Lachnospiraceae_NK4A136_group*, *Prevotellaceae_UCG − 001*, *Helicobacter,* and *Prevotellaceae_UCG − 003* in the model group increased to 16.39%, 7.01%, 5.83%, and 8.81%, respectively, while those in the EPA-treated group decreased to 17.99%, 1.44%, 5.81%, and 3.33%, respectively. These results showed that EPA treatment balanced the relative abundance of beneficial and detrimental bacteria in the gut microflora, as observed in the normal group.

## Discussion

### The antitumor effect of EPA

Chemotherapy is an important cancer treatment, but it has side effects and high toxicity, which may be harmful to the body. In this study, although CTX had the highest TIR (92.42%), it seemed to damage the spleen and thymus, according to the organ index data. Additionally, the AST and BUN levels of the CTX-treated group were significantly increased, as seen by ELISA, thereby increasing the burden on the kidney and liver^26^.

EPA is mainly composed of ergosterol, ergosterol peroxide, ergosta-4, 6, 8(14), 22-tetraen-3-one, and (22E, 24R)-ergosta-7, 22-dien-3β, 5α, and 6β-triol. This study revealed that EPA had a relatively better antitumor effect and had an obvious protective effect on the liver, kidney, spleen, and thymus compared with CTX treatment. The ELISA results showed that EPA may have an inflammatory response that improves IL-2, IL-6, and IFN-γ levels in the serum to enhance immunity^27^. H&E and TUNEL staining were performed to observe apoptosis in tumor cells and showed the remarkable antitumor efficacy of EPA in H22 tumor-bearing mice was mediated by inducing cell death/apoptosis.

The mitochondria-dependent pathway is one of the classic apoptosis pathways, and the most important players are members of the Bcl-2 family^28^. A decrease in the ratio of Bcl-2 to BAX could determine the expression of apoptotic proteins in related tissues30. After treatment with EPA, BAX expression increased significantly. Bcl-2 expression and the ratio of Bcl-2 to BAX decreased. The immunohistochemical and western blot analyses results were consistent. Moreover, previous studies have reported that TNF-α levels could significantly increase the gene expression of BAX and decrease the gene expression of Bcl-2 in human tumor cells^29^; these results were consistent with the ELISA results for TNF-α in this study. Cleaved Caspase 3 is a key apoptotic protein, and its presence indicated that apoptosis had occurred in the tumor cells in this study^30^. Furthermore, cleaved caspase-3 levels significantly increased after EPA treatment. Therefore, we considered that the antitumor mechanism of EPA involves regulating the expression of Bcl-2 and BAX and activating the caspase family of proteins.

VEGF is a tumor marker and an important growth factor implicated in tumor angiogenesis^31^. In this study, after treatment with EPA, VEGF expression was obviously decreased. The immunohistochemical, ELISA, western blot, and RT–PCR results confirmed that EPA could effectively reduce tumor angiogenesis, promote the expression of apoptotic genes and proteins, and inhibit tumor growth.

### Influence on the gut microbiota of mice

In this study, the gut microbiota in mice and the relative abundance of the main genera were analyzed. It was suggested that EPA could increase the abundance of beneficial microflora, decrease harmful microflora and restore the gut microbiota of tumor-bearing mice. The main harmful microbiota identified in this study included *Prevotella*, *Lachnospirace,* and *Helicobacter*.

*Prevotella* is a harmful organism and a common conditional pathogen that can cause dose-dependent visceral hypersensitivity and endogenous infections in some parts of the body. Both *Lachnospirace_NK4A136_group* and *Helicobacter* are classified as gram-negative anaerobic bacteria that can cause severe inflammation, such as diarrhea, watery stools, intestinal infections, bacteriosis, septicemia, cancer, and cholecystitis, and can cause immune dysfunction, bacterial flora imbalance, and endogenous infection^32^. The abundances of *Prevotella,* *Lachnospirace_NK4A136_group,* and *Helicobacter* were 0.02%, 7.59%, and 0.29%, respectively, in the normal group; 16.01%, 23.99%, and 6.12, respectively, in the model group; and 11.23%, 5.99%, and 0.31%, respectively, in the EPA-treated group (Fig. 15 and Table 4). This finding indicated that compared with physiological conditions, the tumor microenvironment can cause an endogenous infection and have an adverse effect on the structure of gut microbiota in mice. However, after treatment with EPA, *Prevotella*, *Lachnospirace_NK4A136_group*, and *Helicobacter* in tumor-bearing mice were significantly reduced and tended to be closer to the levels in the normal group.

**Table 4:** The relative abundance of the main gut microbiota at the genus level in four groups.

**Abundance (%)**			
	**A**	**B**	**C**
*Lactobacillus*	18.075844	11.127102	21.798470
*Bacteroidales_S24-7_group_norank*	28.471373	3.206927	23.148881
*Bacteroides*	6.387470	1.717157	3.058214
*Alloprevotella*	2.667242	0.0959440	3.305270
*Alistipes*	0.702790	0.021587	0.981027
*Lachnospiraceae_NK4A136_group*	7.593965	23.98599	5.994099
*Prevotellaceae_UCG-001*	0.018469	7.195798	5.754240
*Helicobacter*	0.287832	6.115133	0.314217
*Prevotellaceae_UCG-003*	0	8.807656	5.473603
Total	64.204985	62.273294	69.828021

The main beneficial microbiota included *Lactobacillus*, *Bacteroidales*, *Alloprevotella,* and *Alistipes*. *Lactobacillus* is a gram-positive bacterium that regulates the gut microflora, enhances immunity, protects the gastric mucosa, improves intestinal function, prevents diarrhea, promotes digestion and exerts antitumor and antioxidative effects^33,34^. This organism breaks down polysaccharides for other bacteria in the intestine and plays an important role in intestinal balance^35^.

*Bacteroides* and *Alistipes* belong to the genus Bacterioa. *Bacteroides* is a symbiotic bacterium in the gut of humans that provides essential nutrients and directly inhibits the adhesion and invasion of other harmful bacteria through their own colonization^36^. According to reports, *Bacteroides* and its metabolites are closely related to tumors and can regulate cell proliferation, differentiation, and apoptosis and inhibit tumors^37^. *Alistipes* is a relatively new genus that is highly associated with dysbiosis and disease^38^. This genus also has protective effects against some diseases, such as cancer, liver fibrosis, colitis and cardiovascular disease^39^. In this study, EPA effectively reduced tumor angiogenesis and promoted the expression of apoptotic genes and proteins, such as *BAX*, *Bcl-2*, *VEGF*, and cleaved caspase 3. Thus, we can conclude that the antitumor effect of EPA may be related to beneficial microbiota *Bacteroides* and some antitumor or apoptosis pathways.

*Alloprevotella* has the ability to produce folic acid and vitamin B1, which are important bioactive substances that are decomposed and utilize proteins in the intestinal mucosa^40^. The final metabolites are succinic acid and acetic acid. It has been reported that appropriate proportions of succinic acid and acetic acid can directly provide energy for the intestinal epithelium, improve intestinal digestion and absorption of nutrients, and improve intestinal immunity^41^.

## Conclusions and expectation

According to the literature, extracts of edible and medicinal fungi have anti-inflammatory effects and reduce the release of cytokines^42^. In a previous study, polysaccharides were extracted from *Pholiota* *adiposa*, which inhibited tumors^8^. In this study, we concluded that EPA could significantly increase the TIR, especially in the HD group of EPA-treated mice, which was the positive treatment group (CTX). Verification of the molecular mechanism after treatment with EPA by immunohistochemical, ELISA, western blot, and RT–PCR assays showed that EPA could effectively reduce tumor angiogenesis, promote the expression of apoptotic genes and proteins, and inhibit tumor growth.

Instability in the gut microbiota is one of the causes of cancer^43^. Dysbacteriosis may make an individual prone to chronic inflammation, and chronic inflammation increases the risk of developing tumors^44^. At present, the possible correlation between the gut microbiota and tumors has been studied. The gut microbiota also has an impact on the generation, development, therapeutic efficacy, and treatment of malignant tumors^45^.

In this study, harmful microbiota were increased, while beneficial microbiota were decreased in mice inoculated with H22 hepatoma cells. After treatment with EPA, the gut microbiota of tumor-bearing mice tended to be closer to that of normal mice. Hence, EPA may increase the abundance of beneficial microbiota and decrease the abundance of harmful microbiota. The findings of our study provide data for future research on disease and microecology.

In conclusion, although EPA can regulate the gut microbiota of tumor-bearing mice, the specific mechanism remains unclear. With the development of macrogenomics, metabonomics, and molecular biology, researchers will have a more specific understanding of the interaction between food or drugs and the gut microbiota. This study will further deepen and expand research on EPA, its antitumor mechanism, and its influence on the gut microbiota.

## Methods and materials

### Materials and equipment

The fruits of *P.* *adiposa* were purchased from Mushroom professional cooperative in Songyuan, Songyuan City, Changchun, China, which cultivate a variety of edible and medicinal mushroom, such as *Pholiota* *adiposa*, *Pholiota* *microspora*, *Pleurotus* *eryngii*, and *Pleurotus* *ostreatus* et al. In this study, the sample of *P.* *adiposa* were identified by Professor Tolgor Bau of Jilin Agricultural University, who is a famous taxonomist in China. And the voucher specimen of *P.* *adiposa* was deposited in the Fungus herbarium of Jilin Agricultural University which is a publicly available herbarium (Specimen Number, JLAU28202). In this study, the collection of *P.* *adiposa*, complied with relevant institutional, national, and international guidelines and legislation.

All other chemical reagents used in this study were analytical grade. The Cyclophosphamide (CTX) for injection was purchased from Shanghai Huili Biotechnology Co., Ltd. Hematoxylin and Eosin (H&E) dye kits were obtained from Nanjing Jiancheng Bioengineering Research Institute (Nanjing, China). TUNEL assay was performed in situ by using the apoptosis detection kit (Roche, Branchburg, NJ, USA) and the DAB detection kit. ELISA kits for mouse, include Interferon-γ (IFN-γ), Interleukin-2 (IL-2), Interleukin-6 (IL-6), Tumor Necrosis Factor-α (TNF-α), and vascular endothelial growth factor (VEGF) ELISA kits were purchased from American R&D Co., Ltd. (Minneapolis, MN, USA). Rabbit polyclonal antibodies against Bcl-2(3498S), Bax(2772S), Caspase3 (9661 s), and mouse monoclonal VEGF(AV202) antibody were purchased from Cell Signaling Technology (Danvers, MA, USA). TripleTOF 5600 + LC/MS/MS (AB SCIEX, America). Agilent 1290 Infinity LC/MS (Agilent, America). 95% ethanol was purchased from Nanjing Chemical Reagents Co., Ltd. (Nanjing, China); acetonitrile, methanol and acetone were purchased from Merck Company (chromatographic grade, Merck Company of Germany). Real-time PCR detector (LC96, Swiss).

### Preparation and LC–MS analysis of P. adiposa extracts (EPA)

Briefly, dried and powdered of *P.* *adiposa* (1000 g) was extracted with 75% aqueous EtOH by ultrasonic treatment. The ultrasound conditions were ultrasonic power of 500 W, ultrasonic temperature of 60 °C, ethanol concentration of 75%, liquid to solid ratio of 37:1 and extraction time of 32 min. Then, EPA was concentrated, freeze-dried. The final crude extract (66.42 g) was stored at − 20 °C for further use.

For LC–MS analysis, 10 mg of EPA was dissolved in 5 ml of formic acid ultra-pure water solution and was filtered through 0.22 μm nylon membrane before LC–MS analysis. Ultrasonic extraction method was used to extract at 60℃ for 30 min, repeated 3 times, centrifuged at 10 000r/min for 5 min, combined with supernatant and sample analysis. The Waters ACQUITY UPLC BEH C18 (100 mm × 2.1 mm, 1.7 μm) column was used for chromatographic analysis in gradient elution mode at the flow rate of 0.3 mL/min and column temperature of 40℃. The mobile phase A was set as ultra-pure water containing 0.1% formic acid, and mobile phase B is acetonitrile, flow rate: 0.3 mL/min, and gradient elution mode is used for chromatographic separation (Table 5). Ionization mode Qualitative analysis mass spectrometry conditions: electrospray ionization (ESI); Negative ion mode and MSE mode were used for full scan analysis. Ion scanning range: m/z 50 ~ 2000; The temperature of dissolvation is 400℃, the flow rate of dissolvation gas is 800L/h; Low energy voltage: 6 eV; High energy voltage 20-50 eV; Ion source temperature: 120℃, capillary voltage: 2.5 kV. After then, UPLC-QTOF-MSE mode was used to collect data, and Waters UNIFI software was used to analyze the results.

**Table 5:** LC/MS-Q-TOF gradient elution program.

Time(min)	Mobile Phase	
	A(%)	B(%)
0	95	5
2	80	5
5	50	20
15	20	50
45	10	95
55	10	95
55.01	95	5
65	95	5

### Antitumor activity in vivo

#### Animals and cell lines

Specific Pathogen Free grade, 6–8 week old male ICR mice that weighted at 20 ± 2 g, were purchased from Liaoning Changsheng Biotechnology Co., Ltd.(Liaoning, China), with Certificate No.: SCXK (Liao) 2019-0001. The mice were supplied with standard laboratory diet and water ad libitum at a temperature 25 ± 2 °C with a 12-h light/ dark cycle (lights on 8:00 AM to 8:00 PM) and all mice were adapted to the environment for one week. And all experimental procedures were strictly in accordance with the Regulations of Experimental Animal Administration issued by the Ethical Committee for Laboratory Animals at Jilin Agricultural University (Permit No. ECLA-JLAU-19036). And I confirm that all methods are reported in accordance with ARRIVE guidelines for the reporting of animal experiments.

The mouse H22-hepatoma cell line was purchased form Jilin Provincial Cancer Hospital (Jilin, China). H22 tumor mice were prepared as described previously, which were maintained in the ascitic form by sequential passages into the peritoneal cavities of male ICR mice^46^.

#### Preparation of tumor-bearing mice and treatment protocol

**Figure 16:**
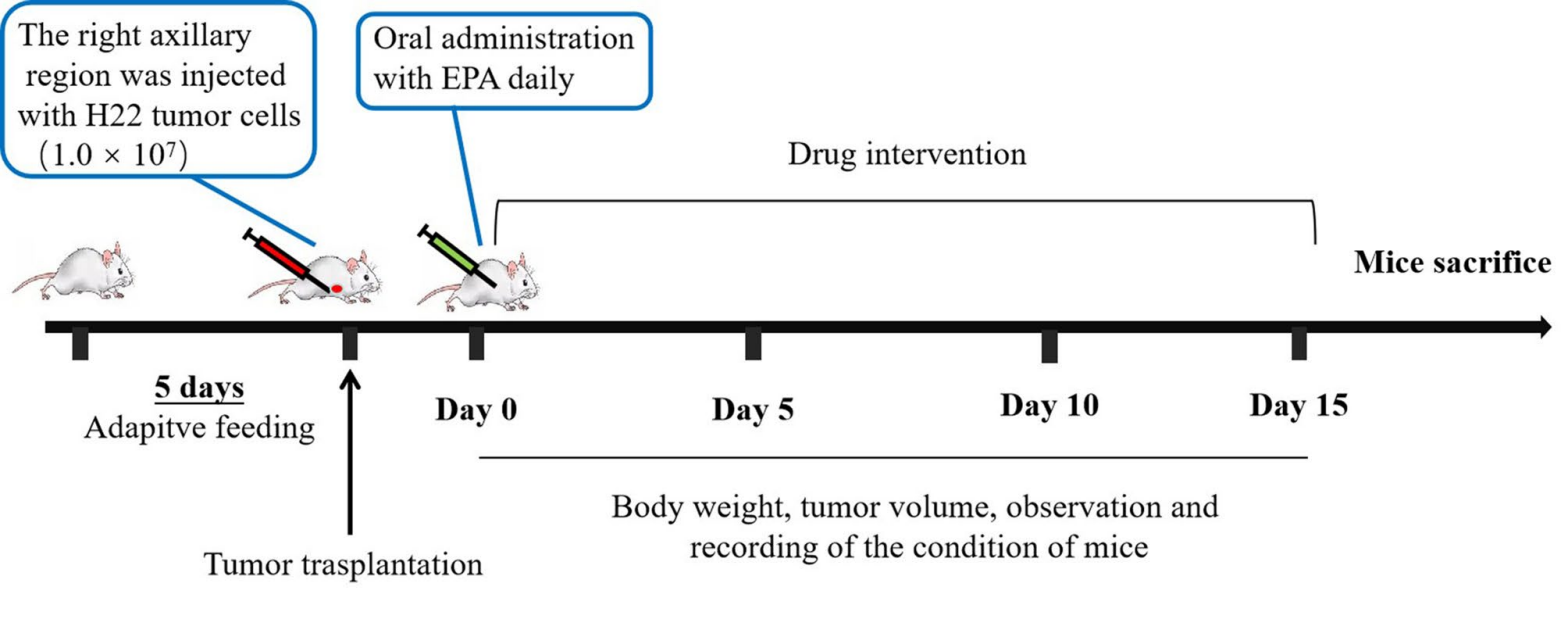
Anti-tumor experimental design scheme of H22 in vivo.

The H22-hepatoma cells was prepared with concentration of 1.0 × 10^7^ cells/mL. All mice except normal group were inoculated in the subcutaneous right forelimb armpit with the tumor cell suspension (0.1 mL for each mice) to establish the tumor-bearing mice model. 24 h later, 10 mice in each group were randomly divided into five groups (The experimental design is shown in Fig. 16) and each 5 mice were in one caged. The tumor-bearing mice were defined as the model group, the positive treatment group and the EPA treatment group, of which the EPA treatment group was further divided into high, medium and low dose groups, the doses of which were 300 mg/kg (HD), 200 mg/kg (MD) and 100 mg/kg (LD) respectively. The control mice were given the same volume of saline solution intragastrically and the positive control were intraperitoneally injected with 25 mg/kg CTX. All groups of mice were treated 1 time/d for 14 days. After then, all mice were killed by euthanasia and the tumor tissues were excised and weighted. The index of tumor inhibition rate (TIR) was calculated as 100% × (average tumor weight of the control group-average tumor weight of the treatment group)/average tumor weight of the control group. The derivative with the highest TIR continued to be subjected to subsequent experiments.

#### Determination of biochemical parameters

Freshly heparinized blood was prepared from orbital venous plexus of the mice. After being centrifuged at 1500 rpm for 20 min at 4 °C, sera were collected and the serum samples of all groups were kept 4℃ for biochemical assay. In accordance with the procedures described in the commercial kit, AST, BUN, IL-2, IL-6, TNF-α, IFN-γ and VEGF levels of the mice were determined by indirect ELISA assay^47^.

#### H & E and TUNEL assay

The H&E staining of H22 solid tumor cells were conducted following the manufacturer's instructions in respective kit, and their results were obtained by optical microscope, and the H & E staining results of the tumor was shown as color images at 400 × magnification^48^.TUNEL assay method was used to detect apoptotic cells in tumor tissues^49^. The tumor tissue sections were treated with 20 mg/mL of proteinase K distilled water for 15 min at room temperature. After that, 50 μL TUNEL solution was added and incubated in the kit at 37℃ for 60 min. Peroxidase activity in each tissue section was indicated by applying diaminobenzidine.

#### Immunohistochemistry

The tumor tissue was fixed in 10% formaldehyde solution for 24 h, then decolorized and buried. After then the tumor tissue were cut into 4-micron slices for immunohistochemical staining which was performed using a murine/rabbit IgG immunohistochemistry kit (VEGF, Bcl-2, BAX,caspase3). Tumor tissue growth and apoptosis were evaluated by cell sequence, size of necrotic area, positive cell count and staining intensity score^50^.

#### Western blot assay and quantitative real-time PCR

The method of western blotting was used for qualitative and quantitative analysis of proteins^51^. Cell lysates were prepared, after then, concentrations of total protein were measured using a BCA assay kit (Pierce, Rockford, IL). Then, the total protein in some tumor tissues was extracted and the protein content of each cell sample was determined by coomasant bright blue method^52^. All proteins were resolved on a 15% SDS-denaturing polyacrylamide gel and then transferred onto a polyvinylidene fluoride (PVDF) membrane. The membranes were incubated with antibodies against Bax(1:1000), caspase3(1:1000), BCl-2(1:1000), and VEGF(1:1000) overnight at 4 °C. The membrane was then washed in PBST and incubated with horseradish peroxidase-conjugated sheep anti-rabbit (1:3000) and equine anti-mouse at room temperature for 1 h. And finally, the enhanced chemiluminescence (ECL) reagent was added and protein mildness was observed by enhanced chemiluminescence detection system (Amersham Pharmacia Biotechnology, Tokyo, Japan). This method was used to analyze the levels of antiapoptotic factor Bcl-2, BAX, VEGF, and Caspase3.

The method of RT-PCR was carried out as described previously^53^. Using Trizol reagent to isolate the total RNA from the tumor tissue, then reverse transcribed into cDNA. And each cDNA sample was tested using a real-time SYBR Green PCR reagent with specific primers (Table 6). The mRNA expression levels of Bcl-2 (3498S), Bax (2772S), Caspase3 (9661 s), VEGF (AV202) and β-actin were calculated by 2 − ΔΔct method and expressed as a ratio compared to control.

**Table 6:** Primers used in the present study.

Primer	Sequence(5'-3')
Caspase3-F	GCGACTACTGCCGGAGTCT
Caspase3-R	ACCGGTATCTTCTGGCAAGC
BAX-F	GGTTGCCCTCTTCTACTTTGC
BAX-R	GTCCAGCCCATGATGGTTCT
BCL2-F	CTCTCGTCGCTACCGTCGT
BCL2-R	CCGAACTCAAAGAAGGCCAC
VEGF-A-F	TACTGCCGTCCGATTGAGAC
VEGF-A-R	TCCAGGGCTTCATCGTTACA

### DNA extraction and 16 s rDNA high-throughput sequencing

#### Collection and preparation of blood samples

Normal group, model group and HD of EPA (300 mg/kg) group were tested, and QIAamp Fast DNA Stool Kit (50) (Qiagen, Hamburg, Germany) was used to extracted the DNA from the feces of the mice. A KAPA HiFi Hotstart ReadyMix PCR Kit was used for the PCR analysis. Using bar-coded primers 341F (5'-CCTAYGGGRBGCASCAG-3') and 806R (5'-GGACTACNNGGGTATCTAAT-3') to amplify the V3-V4 hypervariable regions of bacterial 16 s rDNA (from 341 to 806) from the extracted DNA, after then, the fluorimeter Qubit3.0 were used to pool, purify and quantify the amplicons^54^. The amplicons were purified and quantified before being sequenced on the HiSeq 2500 PE250 Amplifier sequencing platform (Illumina, San Diego, CA, USA) by a commercial company (BIOPROFILE TECHNOLOGY Technology Co. Ltd., SHANGHAI, China).

#### Statistical analysis

All data were presented as the mean ± standard deviation (S.D.). The significance of the differences was determined by a one-way analysis of variance (ANOVA) followed by the Duncan's test, with a p < 0.05 considered to be significant. Statistical analyses were performed using SPSS version 19.0.

Paired-End sequencing were obtained from Illumina PE250 sequencing. Paired-End Reads grow Reads through the Overlap relationship between Reads, and quality control of the spliced Reads. After then, get the Clean Reads. According to overlap, sequence quality is controlled and filtered. OTU cluster analysis (Operational Taxonomic Units) and taxonomic analysis were performed after sample distinguished. Finally, all Clean Reads are compared to OTU, and the Reads of OTU that can be compared are extracted. Get the final apped Reads.

In this research, alpha diversity index analysis method, which reflect the abundance and diversity of microbial communities, was used to analysis the single sample diversity which reflect the abundance and diversity of microbial communities. The diversity of gut bacteria were calculated by Shannon index, Simpson index and PD whole tree index, and abundance of bacteria calculate were calculated by the observed species index and Chao1 index. Beta diversity reflects the differences among different grouping samples. Anosim analysis, PCoA analysis, LefSe difference analysis, rank between groups and other methods were used to analyze the difference significance.

## Supplementary Information

Supplementary Information 1.

Supplementary Information 2.

Supplementary Information 3.

Supplementary Information 4.

Supplementary Information 5.

Supplementary Information 6.

Supplementary Information 7.

Supplementary Information 8.

Supplementary Information 9.

Supplementary Information 10.

Supplementary Information 11.

Supplementary Information 12.

Supplementary Information 13.

Supplementary Information 14.

Supplementary Information 15.

Supplementary Information 16.

Supplementary Information 17.
