# Supplementary figures and images for: Influence of Pholiota adiposa on gut microbiota and promote tumor cell apoptosis properties in H22 tumor-bearing mice
Source: Sci Rep. 2022 May 21;12:8589. doi: 10.1038/s41598-022-11041-x (PMC9124200; doi:10.1038/s41598-022-11041-x)

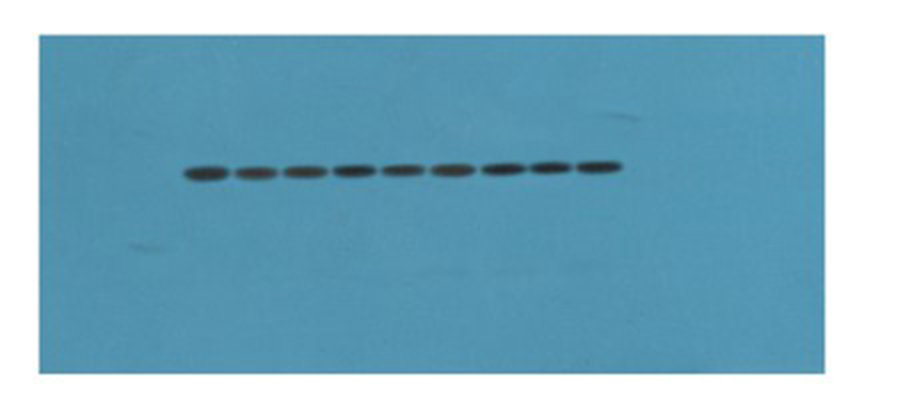

Supplement: Supplementary file 1 — Supplementary Information 1. [file 41598_2022_11041_MOESM1_ESM.jpg]

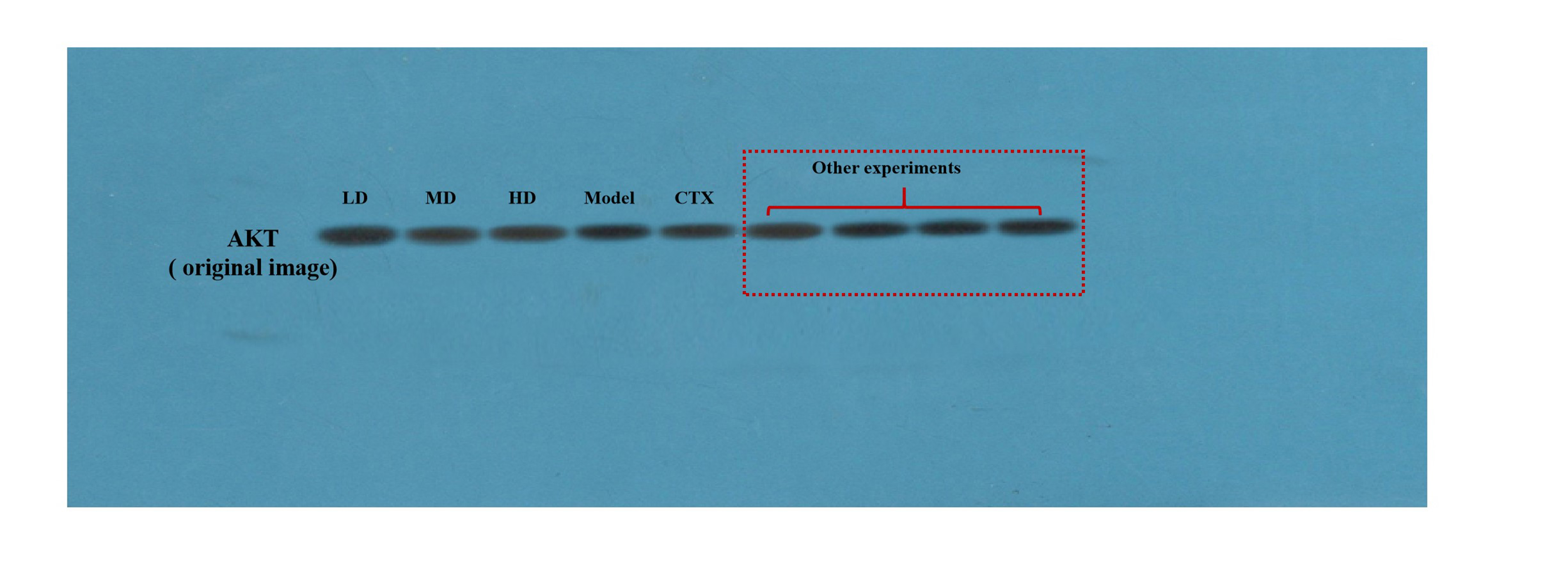

Supplement: Supplementary file 2 — Supplementary Information 2. [file 41598_2022_11041_MOESM2_ESM.jpg]

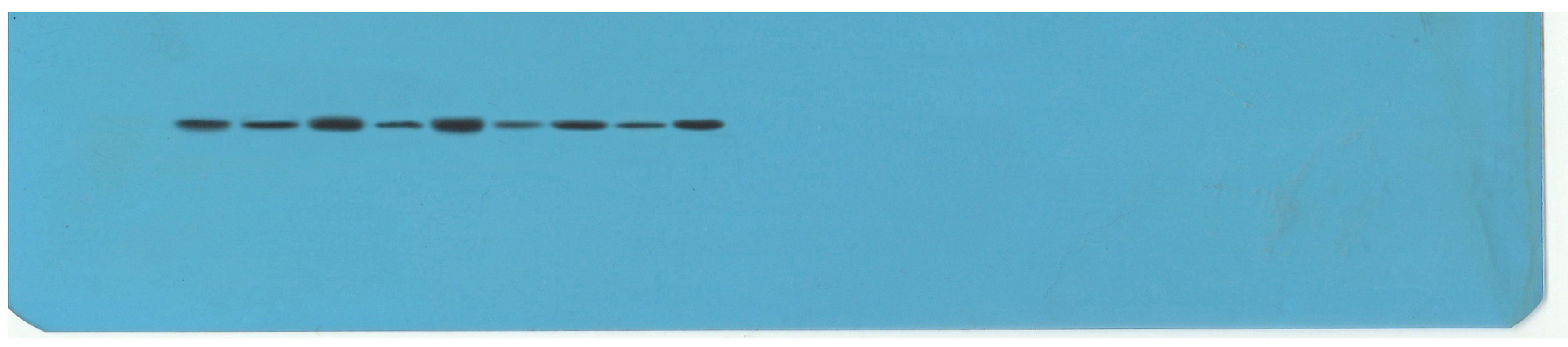

Supplement: Supplementary file 4 — Supplementary Information 4. [file 41598_2022_11041_MOESM4_ESM.jpg]

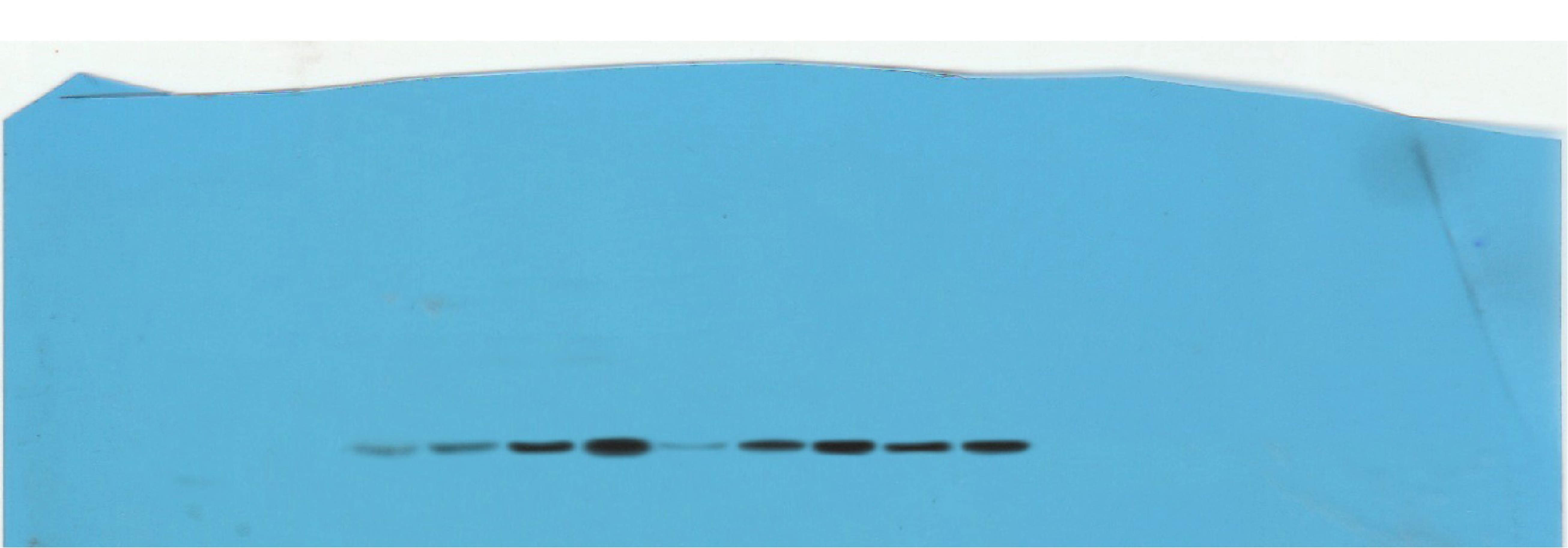

Supplement: Supplementary file 5 — Supplementary Information 5. [file 41598_2022_11041_MOESM5_ESM.jpg]

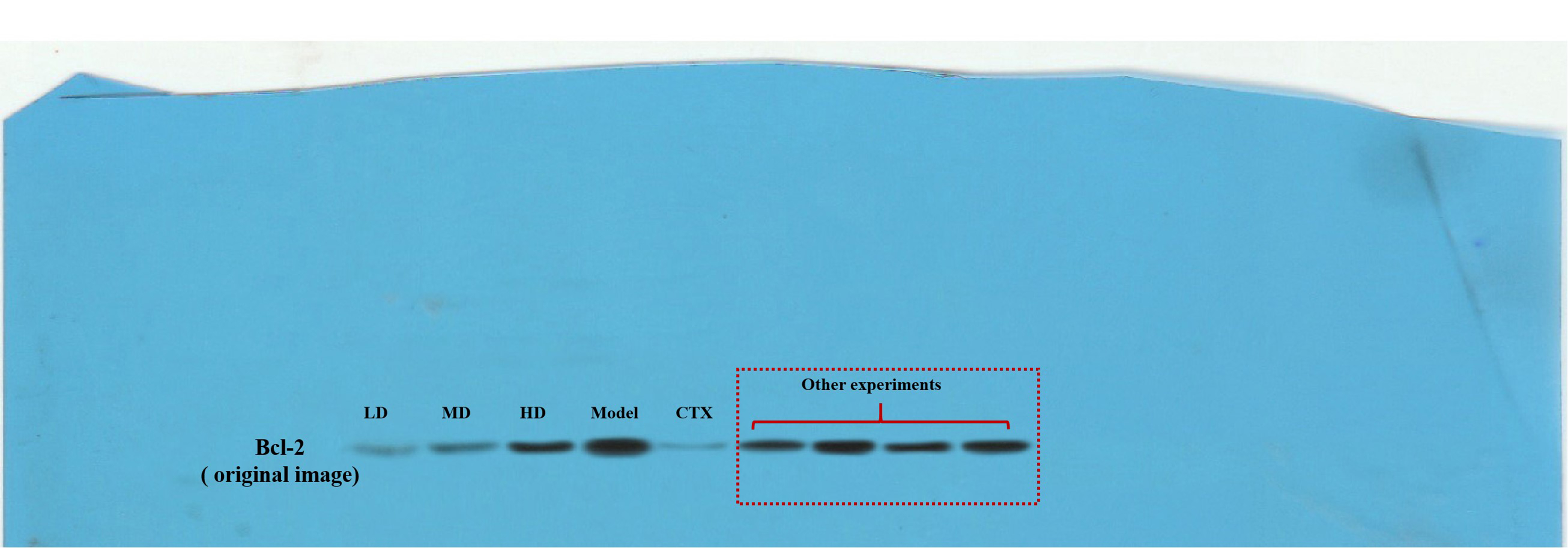

Supplement: Supplementary file 6 — Supplementary Information 6. [file 41598_2022_11041_MOESM6_ESM.jpg]

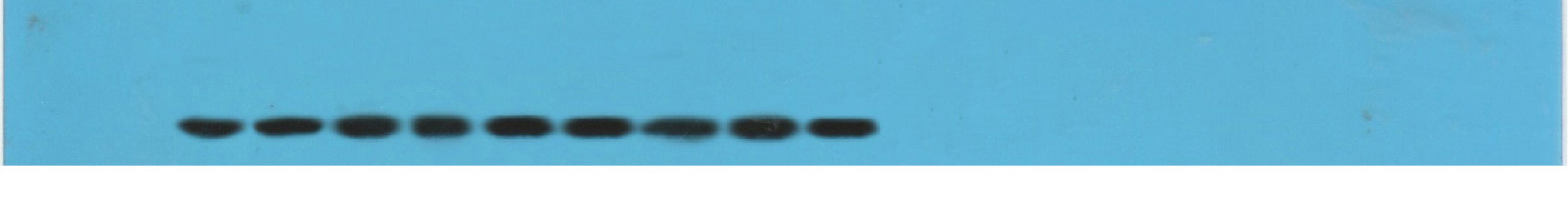

Supplement: Supplementary file 7 — Supplementary Information 7. [file 41598_2022_11041_MOESM7_ESM.jpg]

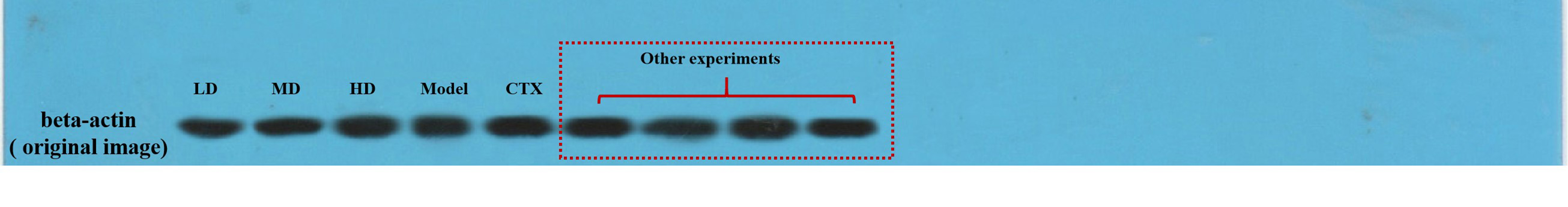

Supplement: Supplementary file 8 — Supplementary Information 8. [file 41598_2022_11041_MOESM8_ESM.jpg]

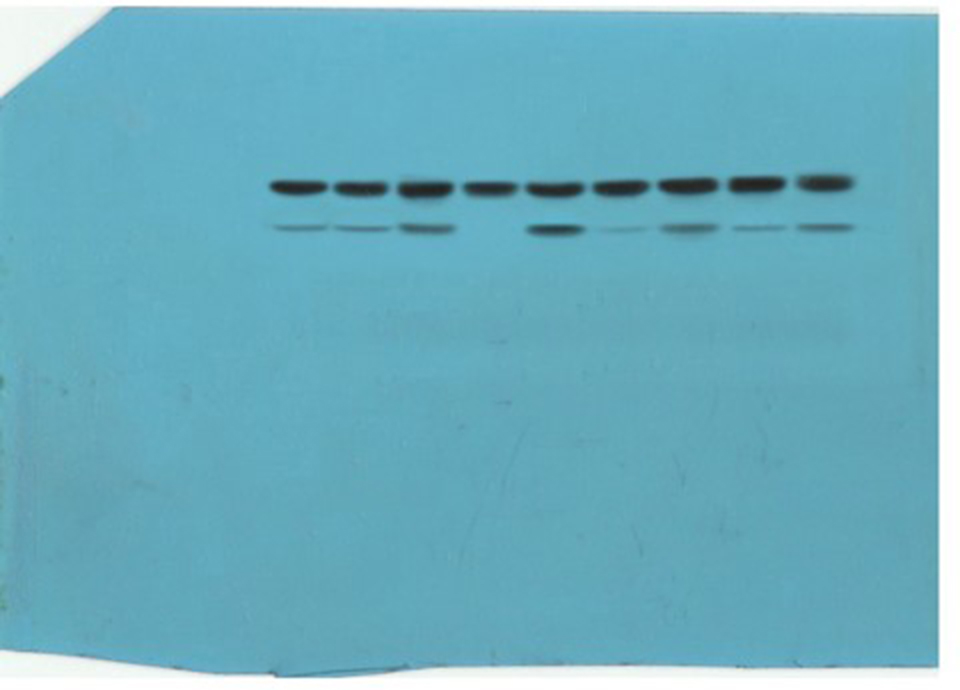

Supplement: Supplementary file 9 — Supplementary Information 9. [file 41598_2022_11041_MOESM9_ESM.jpg]

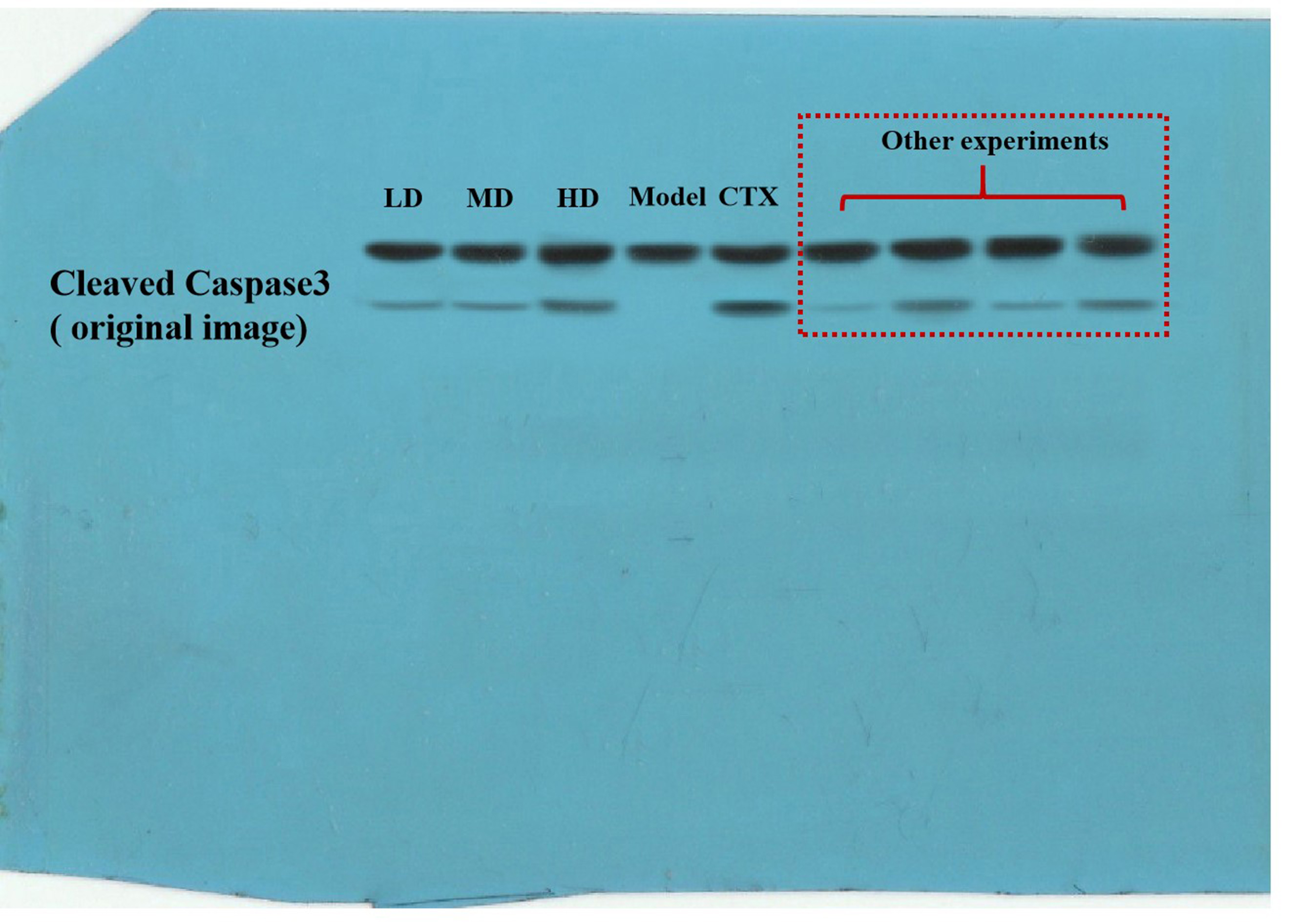

Supplement: Supplementary file 10 — Supplementary Information 10. [file 41598_2022_11041_MOESM10_ESM.jpg]

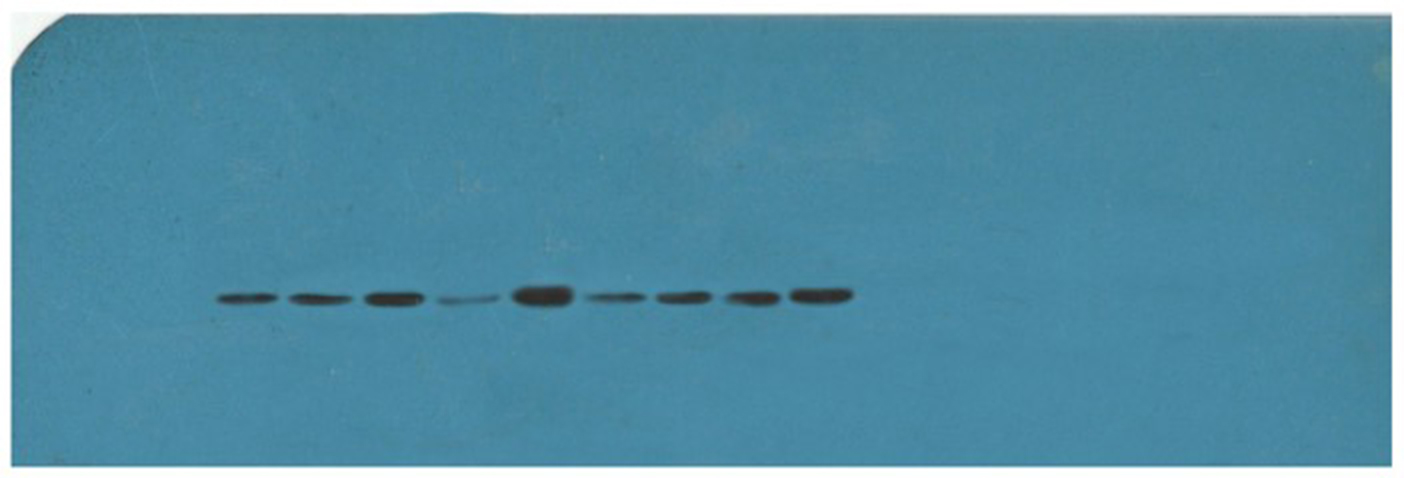

Supplement: Supplementary file 12 — Supplementary Information 12. [file 41598_2022_11041_MOESM12_ESM.jpg]

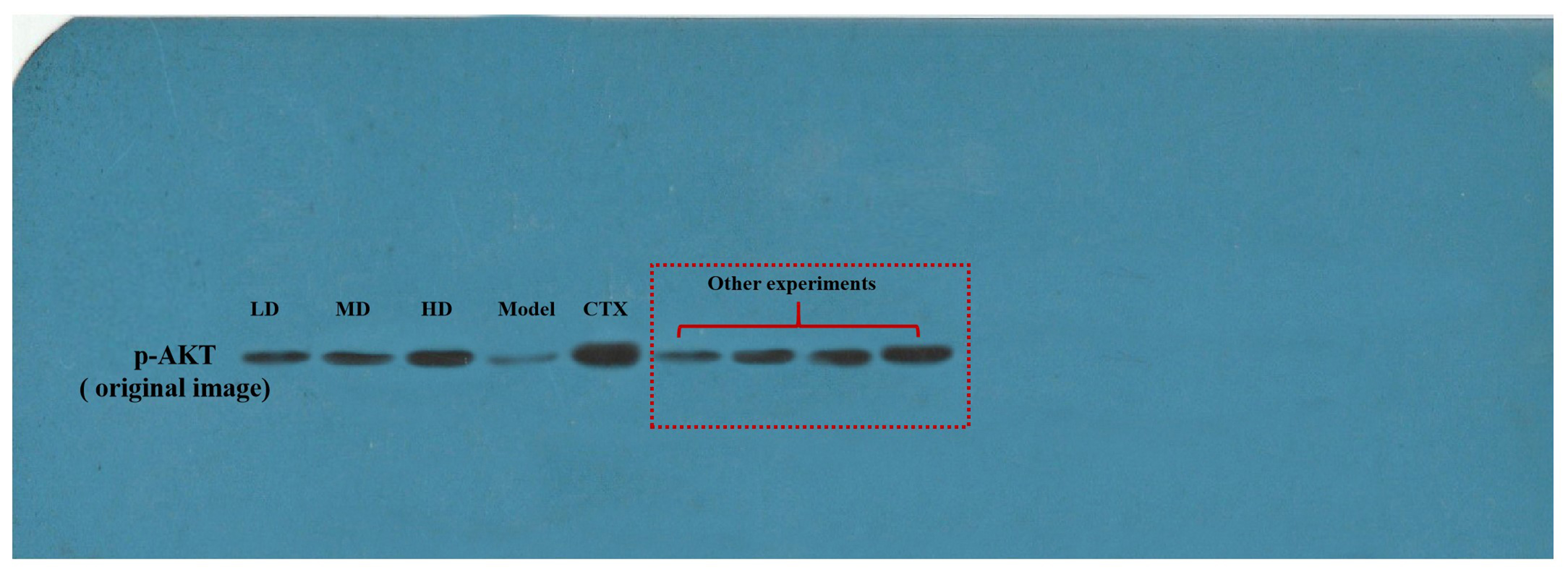

Supplement: Supplementary file 13 — Supplementary Information 13. [file 41598_2022_11041_MOESM13_ESM.jpg]

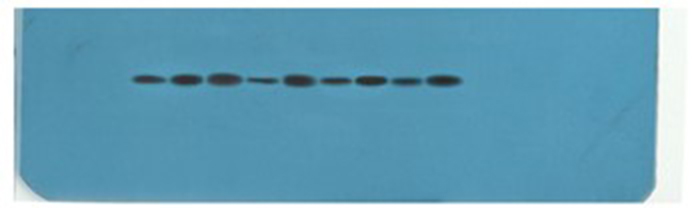

Supplement: Supplementary file 14 — Supplementary Information 14. [file 41598_2022_11041_MOESM14_ESM.jpg]

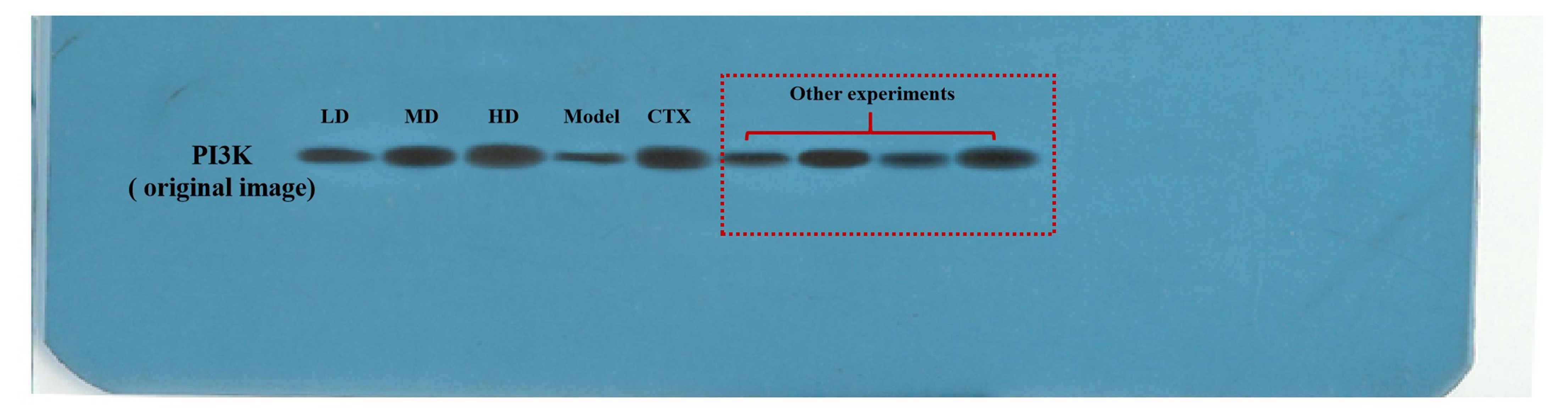

Supplement: Supplementary file 15 — Supplementary Information 15. [file 41598_2022_11041_MOESM15_ESM.jpg]

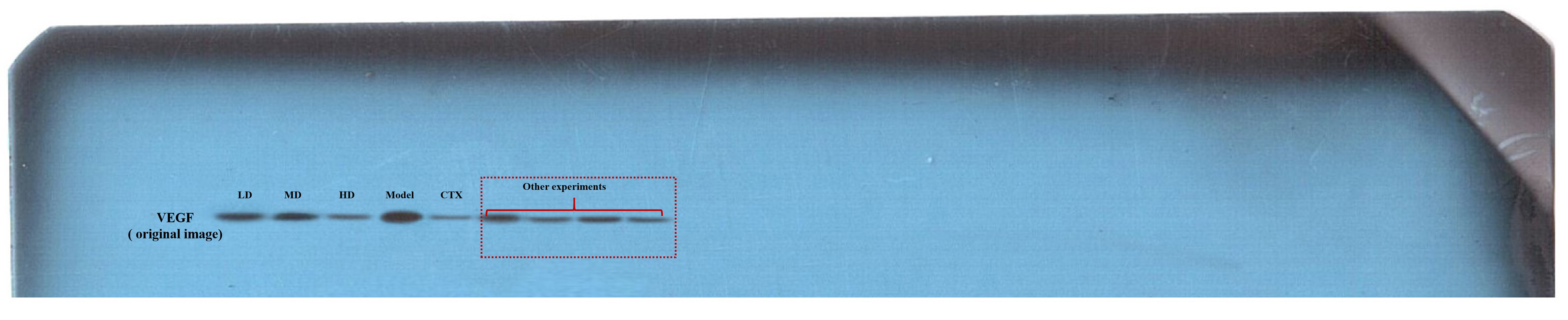

Supplement: Supplementary file 17 — Supplementary Information 17. [file 41598_2022_11041_MOESM17_ESM.jpg]
