# Supplementary material for: Influence of Pholiota adiposa on gut microbiota and promote tumor cell apoptosis properties in H22 tumor-bearing mice
Source: Sci Rep. 2022 May 21;12:8589. doi: 10.1038/s41598-022-11041-x (PMC9124200; doi:10.1038/s41598-022-11041-x)
Supplement: Supplementary file 3 — Supplementary Information 3. [file 41598_2022_11041_MOESM3_ESM.doc]

**Animal experiments**

Specific Pathogen Free grade, 6-8 week old male ICR mice that weighted at 20±2g, were purchased from Liaoning Changsheng Biotechnology Co., Ltd.(Liaoning, China), with Certificate No.: SCXK (Liao) 2019-0001. The mice were supplied with standard laboratory diet and water ad libitum at a temperature 25 ± 2 °C with a 12-h light/ dark cycle (lights on 8:00 AM to 8:00 PM) and all mice were adapted to the environment for one week. And all experimental procedures were strictly in accordance with the Regulations of Experimental Animal Administration issued by the Ethical Committee for Laboratory Animals at Jilin Agricultural University (Permit No. ECLA-JLAU-19036). And I confirm that all methods are reported in accordance with ARRIVE guidelines for the reporting of animal experiments.
