# Supplementary material for: Influence of Pholiota adiposa on gut microbiota and promote tumor cell apoptosis properties in H22 tumor-bearing mice
Source: Sci Rep. 2022 May 21;12:8589. doi: 10.1038/s41598-022-11041-x (PMC9124200; doi:10.1038/s41598-022-11041-x)
Supplement: Supplementary file 16 — Supplementary Information 16. [file 41598_2022_11041_MOESM16_ESM.pdf]

Statement

All experimental procedures were strictly in accordance with the Regulations of Experimental Animal Administration issued by the Ethics committee for Laboratory Animals at the Jilin Agricultural University (Permit No. ECLA-JLAU-19036). And the statement in the attachment. Institution, Ethics committee for Laboratory Animals of the Jilin Agricultural University. And all experiments were performed in accordance with relevant guidelines and regulations. Statement, the reporting in the manuscript follows the recommendations in the ARRIVE guidelines of PLoS Bio 8(6), e1000412,2010.
