# Supplementary material for: Influence of Pholiota adiposa on gut microbiota and promote tumor cell apoptosis properties in H22 tumor-bearing mice
Source: Sci Rep. 2022 May 21;12:8589. doi: 10.1038/s41598-022-11041-x (PMC9124200; doi:10.1038/s41598-022-11041-x)
Supplement: Supplementary file 11 — Supplementary Information 11. [file 41598_2022_11041_MOESM11_ESM.pdf]

About the contrast of WB

In this research, the high-contrast is unavoidable in the result of the western blot.

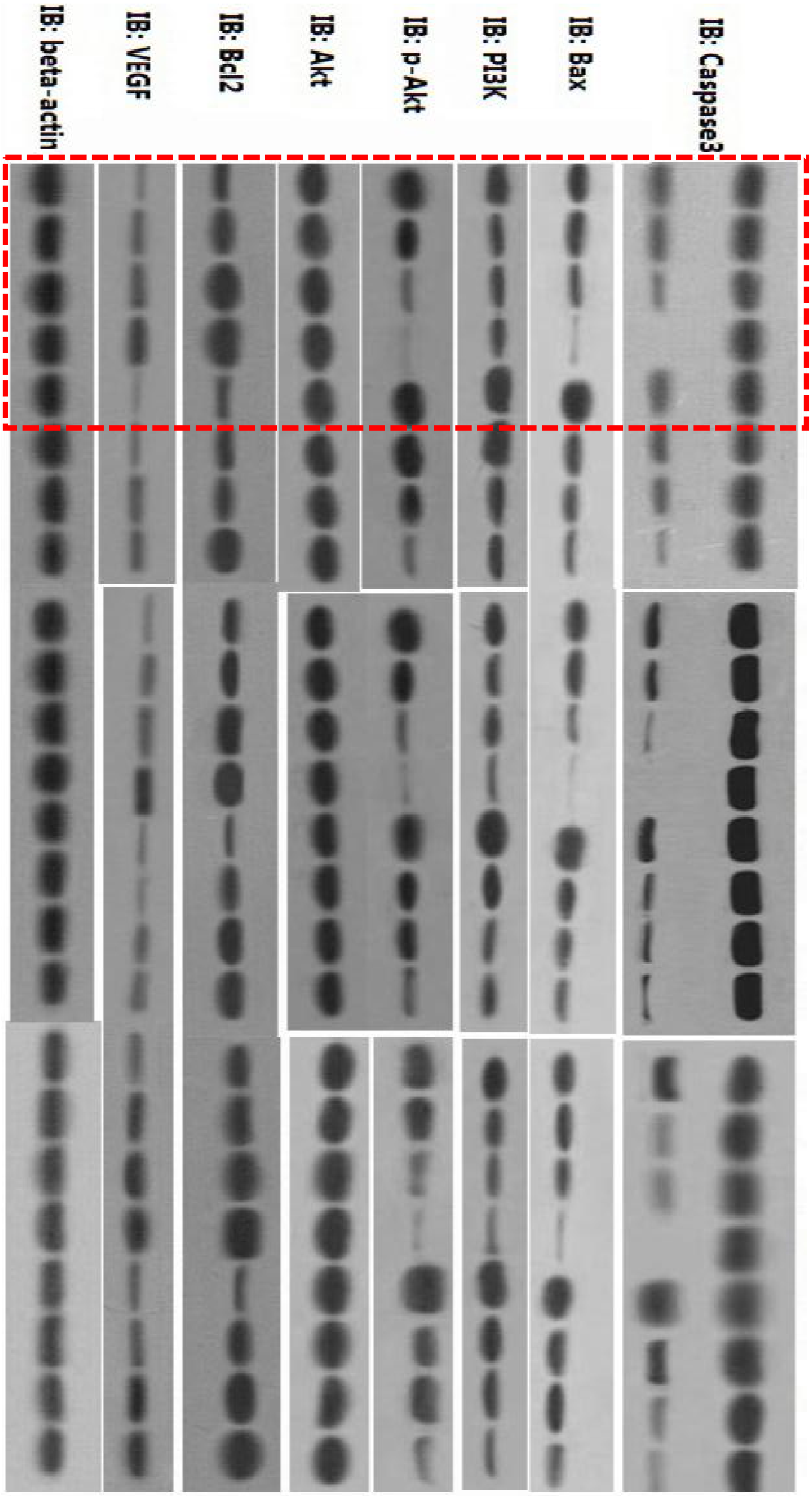

Dear editor, the picture shows the original photo of western blot. The part of this experiment has been clearly marked on the original picture to ensure the authenticity of the experiment.
